# Sonic hedgehog specifies flight feather positional information in avian wings

**DOI:** 10.1242/dev.188821

**Published:** 2020-05-05

**Authors:** Lara Busby, Cristina Aceituno, Caitlin McQueen, Constance A. Rich, Maria A. Ros, Matthew Towers

**Affiliations:** 1 Department of Biomedical Science, University of Sheffield, Western Bank, Sheffield S10 2TN, UK; 2 Instituto de Biomedicina y Biotecnología de Cantabria, IBBTEC (CSIC-Universidad de Cantabria - SODERCAN), 39011 Santander, Spain; 3 Departamento de Anatomía y Biología Celular, Facultad de Medicina, Universidad de Cantabria, 39011 Santander, Spain

**Keywords:** Avian, Chick, Embryo, Flight feather, Positional information, Shh

## Abstract

Classical tissue recombination experiments performed in the chick embryo provide evidence that signals operating during early limb development specify the position and identity of feathers. Here, we show that Sonic hedgehog (Shh) signalling in the embryonic chick wing bud specifies positional information required for the formation of adult flight feathers in a defined spatial and temporal sequence that reflects their different identities. We also reveal that Shh signalling is interpreted into specific patterns of *Sim1* and *Zic* transcription factor expression, providing evidence of a putative gene regulatory network operating in flight feather patterning. Our data suggest that flight feather specification involved the co-option of the pre-existing digit patterning mechanism and therefore uncovers an embryonic process that played a fundamental step in the evolution of avian flight.

## Abstract (precis)

**Highlighted Article:** Embryonic Shh signalling integrates flight feather with digit specification in the chicken wing, providing insights into the evolution of avian flight.

## INTRODUCTION

Although much is known about the molecular pathways involved in the induction, positioning and morphogenesis of feathers (Chen et al., 2015; Chang et al., 2019), little is known about how different types of feathers are specified. Classical tissue recombination experiments in chickens provide evidence that signals acting at the earliest stages of wing bud development (day 3.5 of incubation or HH20 - see Materials and Methods for staging) specify feather identity (Cairns and Saunders, 1954; Saunders and Gasseling, 1959). Thus, grafts of prospective chick thigh mesoderm made to the wing result in the formation of feathers characteristic of those found in the leg. These findings show that the cells of the morphologically indistinct wing bud mesoderm, which give rise to the dermis, have non-equivalence (have a different intrinsic character), and thus carry positional information that determines feather identity in the overlying ectoderm (Lewis and Wolpert, 1976).

**Fig. 1.**
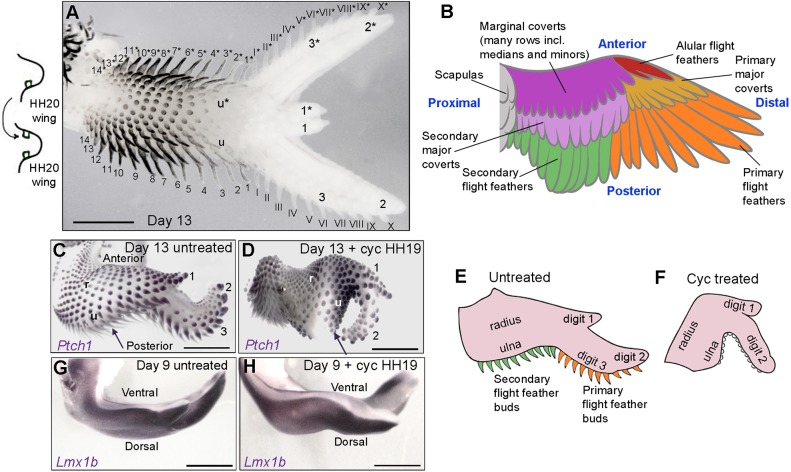
**Shh is required for flight feather bud formation.** (A) Polarising region grafts made to the anterior margin of host chick wing buds at HH20 duplicate all tissues across the antero-posterior axis at day 13, including the digits and feather buds (asterisks show duplicated tissues; Roman numerals; primary flight feather buds; Arabic numerals; secondary flight feather buds; black is feather pigmentation). (B) Schematic showing general bird wing feather pattern, including the three types of flight feathers: primaries along the posterior margin of digits 2 and 3; secondaries along the posterior margin of the ulna; and alulars along the posterior margin of digit 1 (Lucas and Stettenheim, 1972). The chicken has 10 primary and 18 secondary flight feathers, a row of primary and secondary major coverts, and many rows of marginal coverts of different identities, including median and minor coverts. (C,D) Developing flight feathers, as shown by *Ptch1* expression in all buds in untreated day 13 wings (C, arrow) but not in wings of embryos treated with cyclopamine (cyc) at HH19 (D, arrow; *n*=4/4). Treatments at HH19 often result in loss of digit 3, but not the radius (r) or ulna (u) (Towers et al., 2011). (E) Schematic depicting the flight feather bud pattern shown in C. (F) Schematic depicting posterior feather bud pattern shown in D. (G,H) *Lmx1b* expression in the dorsal mesendoderm of untreated (G; *n*=4/4) and HH19 cyclopamine-treated (H; *n*=4/4) wing buds at day 9. Scale bars: 2 mm in A,C,D; 1 mm in G,H.

An important signal known to operate at HH20 is Sonic hedgehog (Shh) – a protein produced by a transient signalling centre called the polarising region (also known as the zone of polarising activity or ZPA), which is located in mesoderm at the posterior margin of the limb bud (Riddle et al., 1993). Shh is implicated in the specification of antero-posterior positional values (thumb to little finger, digits 1, 2 and 3) in chick limb bud cells derived from the lateral plate mesoderm in a concentration-dependent manner between HH18 and HH22 (Yang et al., 1997; Towers et al., 2011). Shh is also involved in stimulating proliferative growth along the antero-posterior axis (Towers et al., 2008; reviewed by Tickle and Towers, 2017). Grafts of *Shh*-expressing polarising region cells made to the anterior margin of host HH20 wing buds (day 3.5) duplicate the antero-posterior axis to produce digit patterns such as 3, 2, 1, 1, 2 and 3 Fig. 1A; Tickle et al., 1975; Riddle et al., 1993). Other tissues that are not derived from the lateral plate mesoderm, including the nerves and muscles, are duplicated as a secondary consequence (Luxey et al., 2020), and thus show equivalence (Lewis and Wolpert, 1976; i.e. progenitor cells are not intrinsically different in character and do not carry positional information). The pattern of feather buds is also duplicated across the antero-posterior axis (Fig. 1A,B; see also Riddle et al., 1993). [Secondary flight feather buds are marked in Arabic numerals, primaries in Roman numerals and alulars are yet to form.] Therefore, the fact that, like the digit skeleton and other connective tissues, the dermis originates from multipotent lateral plate mesoderm progenitor cells (Pearse et al., 2007) raises the possibility that it is also specified with positional values in response to Shh signalling, and that this could determine feather identity. Alternatively, feathers could be specified independently of antero-posterior polarity or by other signals.

In this study, we show that Shh signalling by the embryonic chick wing polarising region specifies the pattern of flight feathers, which provide most of the flapping, gliding and soaring ability required for airborne locomotion in birds (Matloff et al., 2020). Our data provide evidence that Shh signalling integrates the patterning of digits and flight feathers, and thus provides insights into the co-evolution of these important structures.

## RESULTS

### Shh signalling is required for flight feather bud formation

To determine whether Shh signalling specifies feather identity in the chick wing, we transiently inhibited it using cyclopamine at day 3 of incubation (HH19) for ∼72 h (Pickering and Towers, 2016). Cyclopamine is a potent inhibitor of the Hedgehog signalling pathway at the level of Smoothened (Taipale et al., 2000). When cyclopamine is systemically applied to chick embryos at HH18-20, it causes the loss of posterior structures in both wings and legs (Scherz et al., 2007; Towers et al., 2008, 2011) and closely mimics the genetic inactivation of Shh signalling in chicken *oligozeugodactyly* mutants (Ros et al., 2003). At day 13 of development, we observed abnormal flight feather bud development. Thus, elongated flight feather buds expressing *Ptch1* [a direct target of Shh signalling that is involved in feather morphogenesis (Harris et al., 2002, 2005; McKinnell et al., 2004)] are found along the posterior margin of untreated day 13 wings (arrow in Fig. 1C, schematic shown in E), but not in the wings of embryos that were treated with cyclopamine (Fig. 1D). Here, the feather buds along the posterior margin are identical to those in other places, suggesting that they are not flight feathers (arrow in Fig. 1D). The schematic representation in Fig. 1E,F shows how the feather buds along the posterior border of the cyclopamine-treated wing do not show the typical elongated morphology.

In addition to its function in limb patterning, Shh is also involved in the epithelio-mesodermal interactions that drive feather formation (Harris et al., 2002, 2005; McKinnell et al., 2004). However, the observation that *Ptch1* is still expressed in feather buds of wings treated at HH19 with cyclopamine (Fig. 1D) suggests that it is the earlier loss of Shh signalling by the polarising region that prevents flight feather bud formation, rather than the loss of Shh signalling within the buds themselves.

The failure of flight feather bud formation could be interpreted as a secondary consequence of the loss of all posterior tissues – e.g. digit 3 often does not form in wing buds treated with cyclopamine at HH19 (Fig. 1D; Towers et al., 2011). This is an important consideration because, in the case of the muscles, their absence would be due to the loss of migrating myoblasts into posterior regions of the wing. However, wing bud mesoderm, which differentiates into the dermis, is not lost following cyclopamine exposure, but instead contributes to structures that are anteriorised (i.e. cells that would have contributed to digit 3 now contribute to digit 2) (Towers et al., 2011, 2008). In addition, flight feather buds often fail to form in forewing regions that have no overt changes in antero-posterior patterning (which always form a radius and ulna, Fig. 1D,F).

Flight feather buds form along the dorsal-ventral boundary of the wing, which, when disrupted, can result in abnormal flight feather development (Grieshammer et al., 1996). Therefore, to examine whether the loss of Shh signalling affects dorso-ventral patterning of the wing bud, we examined the expression of *Lmx1b*, which is expressed in the dorsal mesoderm. In both the wing buds of untreated (Fig. 1G) and HH19 cyclopamine-treated embryos (Fig. 1H), the expression of *Lmx1b* reveals that the dorsal-ventral boundary remains intact when feather buds initiate development at day 9. Our results indicate that early Shh signalling from the polarising region is required for the later formation of flight feather buds, independently of dorso-ventral polarity.

### Shh is required for flight feather development during late embryogenesis

**Fig. 2.**
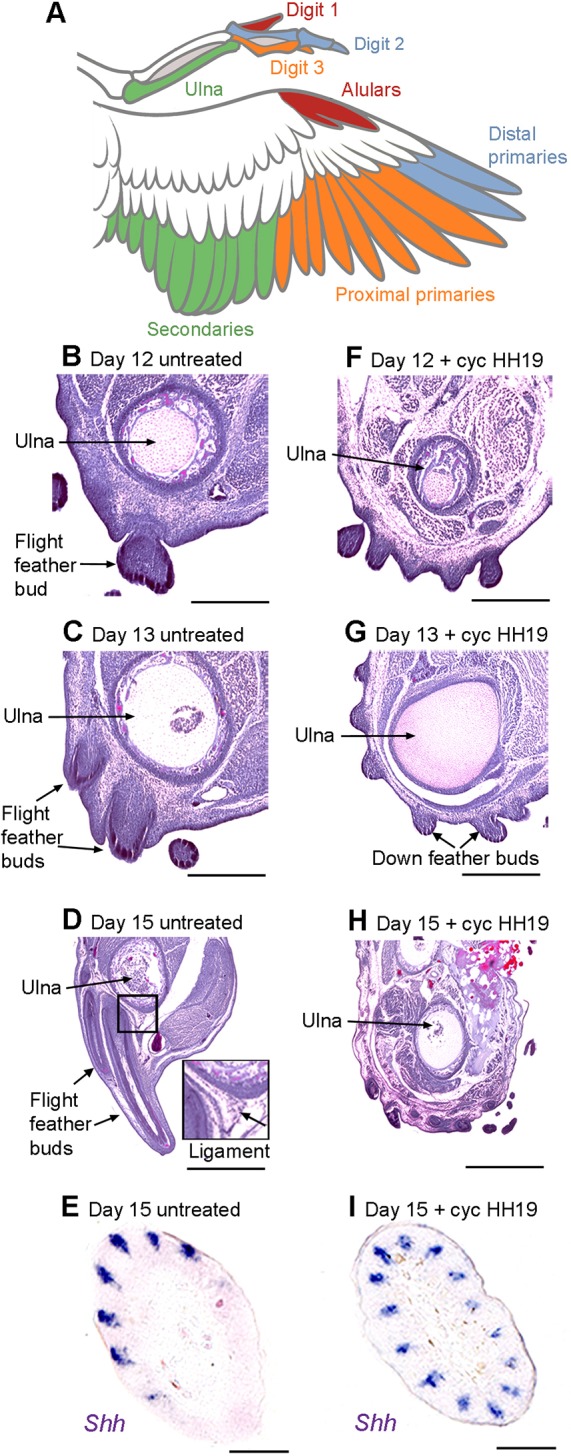
**Shh is required for flight feather development during late embryogenesis.** (A) Schematic showing relationship between flight feathers and the skeleton to which they connect using ligaments. (B-D) Hematoxylin and Eosin staining on transverse sections of day 12 to day 15 forewings showing developing flight feathers in untreated embryos and their connections to the ulna (*n*=15/15). (E) Asymmetric expression of *Shh* in flight feather buds on forewings of untreated embryos (*n*=6/6 wings). (F-H) Absence of flight feathers making ligamentous connections with the ulna in cyclopamine-treated embryos (*n*=18/23). (I) Asymmetric expression of *Shh* is not observed in feather buds on the forewings of cyclopamine-treated embryos (*n*=6/6 wings). Scale bars: 100 μm in B,F; 125 μm in C,G; 150 μm in D,H; 50 μm in E,I.

Developing flight feather buds become morphologically distinct during late embryogenesis by growing inwards to make ligamentous connections with the skeleton, and by displaying bilateral asymmetry (Lucas and Stettenheim, 1972; Kondo et al., 2018). The schematic in Fig. 2A shows the flight feather pattern and its association with the digit skeleton. We used these morphological characteristics to determine whether the feather buds observed in wings that were treated at HH19 with cyclopamine might be retarded in their growth and develop into flight feathers at later stages of embryogenesis, or if they are not truly flight feathers. Hematoxylin and Eosin staining on transverse sections of untreated forewings reveals that flight feather buds grow away from the posterior margin of the wing, and also invaginate into deeper tissues until they reach the ulna by day 13 (Kondo et al., 2018; Fig. 2B-D). In addition, developing flight feather buds can also be identified in transverse section by their asymmetric pattern of *Shh* expression at day 15 (Kondo et al., 2018; Fig. 2E). However, in the wings of embryos treated with cyclopamine, flight feather buds that produce ligamentous connections with the ulna frequently fail to form along the posterior border of the wing. The surface of the wing remains covered with natal down buds, none of which invaginates deeply towards the skeleton (Fig. 2F-H). Furthermore, only developing feather buds with symmetrical expression of *Shh* are observed in forewing regions at day 15, again demonstrating the specific absence of flight feather buds (Fig. 2I). In summary, these observations demonstrate the transient and specific requirement of polarising region-derived Shh signalling for advanced stages of flight feather bud development during late embryogenesis.

### RNA sequencing reveals a flight feather bud transcriptome

Recently, molecular markers of the flight-feather forming regions of the chick wing have been identified, including *Sim1* (Seki et al., 2017) and *Zic1* (Wu et al., 2018). Notably, *Sim1* has an avian-specific forelimb expression pattern in the dermis (Coumailleau and Duprez, 2009; Seki et al., 2017). To determine whether ‘memory’ of exposure to early Shh signalling is interpreted into patterns of flight feather bud-associated gene expression, we performed a series of RNA sequencing (RNA-seq) experiments on tissue dissected from day 10 wings. This stage was selected because it is when flight feather buds become morphologically distinct from other feather buds (see expression of a general marker, *Bmp7*; Michon et al., 2008), in raised flight feather buds at this stage, Fig. S1). We sequenced RNA extracted from soft tissue flanking the posterior margin of the ulna at day 10, because this tissue forms normally in all wings treated with cyclopamine at HH19.

**Fig. 3.**
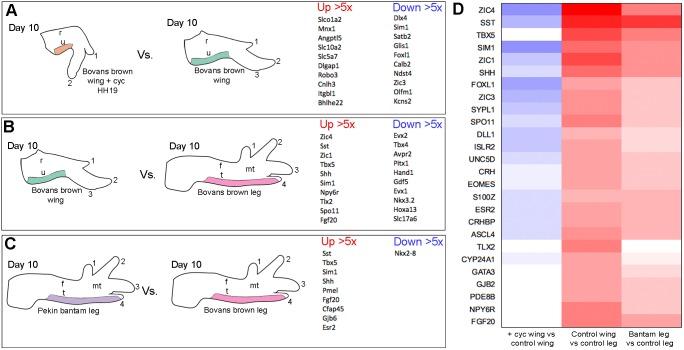
**RNA-sequencing reveals a flight feather bud transcriptome.** (A-C) Schematics showing regions of day 10 limbs that were used to make RNA and the pairwise contrasts made: HH19 cyclopamine-treated Bovans brown wings versus control Bovans brown wings (A), Bovans brown wings versus Bovans brown legs (B) and Pekin bantam legs versus Bovans brown wings (C). The top ten genes up- and downregulated more than fivefold are shown for each comparison (*P*<0.005). (D) Cluster of genes downregulated in wings by earlier Shh signalling inhibition, upregulated in wings versus legs, and upregulated in Pekin bantam legs versus Bovans brown legs (*P*<0.005 and a greater than twofold change in at least one contrast; red, upregulated; blue, downregulated. r, radius; u, ulna; t, tibia; f, fibula; mt, metatarsals.

We contrasted sequencing data from the posterior forewing regions of cyclopamine-treated and untreated control Bovans brown wings (Fig. 3A; the top ten genes up- and downregulated by more than five-fold are shown, see Table S3 for more information). We enriched for genes associated with feather bud development by contrasting RNA-seq datasets obtained from Bovans brown wings with one obtained from the corresponding region of Bovans brown legs, which produce scales instead of feathers (Fig. 3B and Table S3). To further enrich for genes associated specifically with flight feather bud development, we also compared RNA-seq datasets from the posterior soft tissues of Bovans brown legs and Pekin bantam legs (Fig. 3C and Table S3). Pekin bantam legs develop feathers, including flight feathers along their posterior margins (ptilopody), whereas most chicken breeds, including Bovans browns, produce only scales (Prin and Dhouailly, 2004). *Sim1* is found in all three pairwise contrasts at a greater than a fivefold expression difference: downregulated in wings after cyclopamine treatment (Fig. 3A); upregulated in wings versus legs (Fig. 3B); and upregulated in Pekin bantam versus Bovans brown legs (Fig. 3C). These results were confirmed by qPCR performed on cDNA synthesised from the RNA that was used in the sequencing analysis (Fig. S2). We performed a hierarchical clustering analysis to identify genes that behave similarly across the three comparisons (the expression levels of all genes included exhibit a more than twofold difference between at least one contrast, with *P*<0.005). This analysis produced four clusters (Table S3), and we focused on cluster four, which comprises 26 known genes, including *Sim1* and *Zic1* (Fig. 3D).

### Shh signalling is required for flight feather associated gene expression

**Fig. 4.**
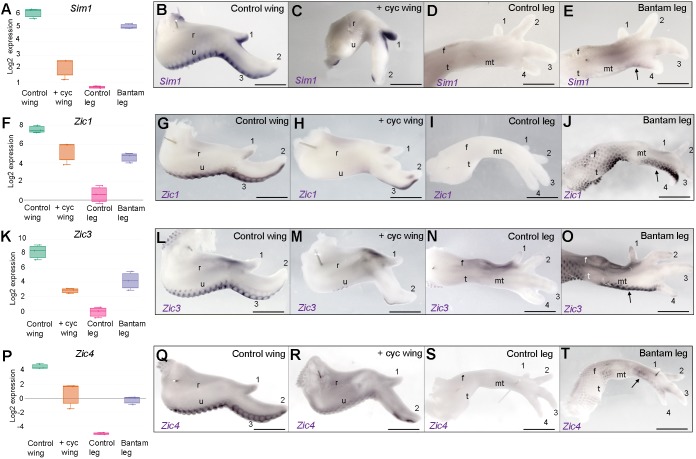
**Shh signalling is required for flight feather bud-associated gene expression.** (A,F,K,P) Box and whisker plots showing relative expression levels of *Sim1* (A), *Zic1* (F), *Zic3* (K) and *Zic4* (P) as normalised log_2_ values of RNA sequencing read-count intensities. (B,G,L,Q) Expression of *Sim1* (B, *n*=22/22), *Zic1* (G, *n*=4/4), *Zic3* (L, *n*=4/4) and *Zic4* (Q, *n*=4/4) in flight feather-forming regions of day 10 wings. (C,H,M,R) Downregulation of *Sim1* (C, *n*=12/14), *Zic1* (H, *n*=2/2), *Zic3* (M, *n*=2/2) and *Zic4* (R, *n*=2/2) in forewing regions following Shh signalling inhibition. (D,I,N,S) Undetectable/weak expression of *Sim1* (D, *n*=2/2), *Zic1* (I, *n*=2/2), *Zic3* (N, *n*=4/4) and *Zic4* (S, *n*=2/2) in Bovans brown legs. (E,J,O,T) Upregulation of *Sim1* (E, *n*=2/2), *Zic1* (J, *n*=2/2), *Zic3* (O, *n*=2/2) and *Zic4* (T, *n*=2/2) along the posterior margins of Pekin bantam legs. For box and whisker plots, centre mark is median, whiskers are minimum/maximum. Arrows indicate ectopic gene expression. Scale bars: 1 mm. t, tibia; f, fibula; mt, metatarsals.

Boxplots of the read-counts obtained from RNA-sequencing data show relative expression levels of *Sim1* and *Zic1* (Fig. 4A,F), and we confirmed their expression patterns in the flight feather-forming regions of day 10 wings (Fig. 4B,G). Their expression patterns are not identical: *Zic1* is only weakly expressed along the posterior margin of digit 1 (Fig. 4G); however, both *Sim1* (Fig. 4C) and *Zic1* (Fig. 4H) are undetectable along most of the posterior margin of the ulna and digit 2 of day 10 wings treated with cyclopamine at HH19, whereas *Sim1* is still observed in digit 1 (Fig. 4C). In addition, although both *Sim1* and *Zic1* are undetectable along the posterior margin of Bovans brown legs (Fig. 4D,I), they are expressed in equivalent regions of Pekin bantam legs (Fig. 4E,J). *Zic3* and *Zic4* are also present in this cluster (Fig. 3D, Fig. 4K,P) and both share very similar expression patterns to *Zic1* in normal wing development (compare Fig. 4G,L and Q). As found with both *Sim1* and *Zic1*, the inhibition of Shh signalling reduces the expression of *Zic3* and *Zic4*, particularly in forewing regions (Fig. 4M,R). Furthermore, compared with the posterior margins of Bovans brown legs, in which *Zic3* and *Zic4* expression is undetectable (Fig. 4N,S), *Zic3* is strongly expressed in Pekin bantam legs (Fig. 4O) and *Zic4* is weakly expressed (Fig. 4T). These results provide evidence for a potential gene regulatory network in flight feather development that interprets the memory of the earlier exposure to Shh signalling.

### The duration of Shh signalling is interpreted into the later spatial pattern of *Sim1* expression

**Fig. 5.**
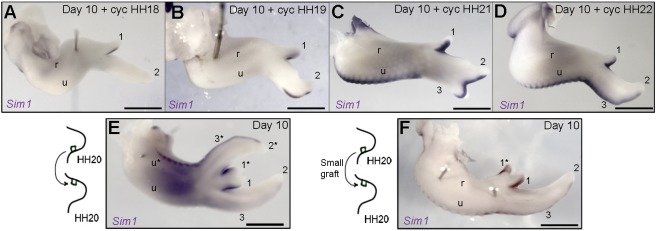
**The duration of Shh signalling is interpreted into the later spatial pattern of *Sim1* expression.** (A-D) Application of cyclopamine at HH18, HH19, HH21 and HH22 reduces *Sim1* expression in digit 1 at day 10 and causes loss of expression in the ulna and digit 2 (A, *n*=3/3), reduces *Sim1* expression in the ulna and the proximal region of digit 2 (B, *n*=12/14), (C) reduces *Sim1* expression in digit 3 (C, *n*=10/15) and does not affect *Sim1* expression (D, *n*=5/5). (E) HH20 polarising region grafts made to the anterior margin fully duplicate the pattern of *Sim1* expression (*n*=5/5). (F) Smaller HH20 polarising region grafts made to the anterior margin duplicate *Sim1* expression in the additional digit 1* (*n*=2/2). Scale bars: 1 mm.

*Sim1* appears to be the clearest marker of the flight feather-forming regions of the chick wing from day 8 to day 13 (Fig. 4B and Fig. S3) – compare this with *Bmp7* expression in all feather buds (Fig. S1). In order to precisely define the temporal requirement for Shh signalling in specifying the later pattern of *Sim1* expression, we applied cyclopamine at different stages. Application at HH18 causes loss of *Sim1* expression along the posterior margin of the ulna and digit 2 of day 10 wings, and significantly reduces expression in digit 1 (Fig. 5A). Progressively later treatments at HH19 cause loss of *Sim1* expression in the ulna and the proximal part of digit 2 (Fig. 5B), and at HH21, loss of expression in digit 3 only (Fig. 5C). In addition, although *Shh* is expressed until HH28 (Riddle et al., 1993), treatment with cyclopamine at HH22 does not affect *Sim1* expression (Fig. 5D).

To determine whether an ectopic source of Shh signalling is sufficient to induce *Sim1* expression, we grafted HH20 polarising regions to the anterior margin of stage-matched host chick wing buds. In day 10 wings, *Sim1* is expressed along the anterior margin of the duplicated skeletal elements (asterisks in Fig. 5E), forming a mirror image of its normal pattern (Fig. 4B). Furthermore, we also performed smaller polarising region grafts to reduce the concentration of Shh signalling (see Tickle, 1981). In day 10 wings, *Sim1* expression is specifically duplicated along the anterior margin of the additional digit 1 (asterisk, Fig. 5F). However, although it is expected that flight feather buds would be duplicated, alular feather buds have still not formed at day 14 (see Fig. 1A) and, owing to the nature of the experiments, we could not obtain ethical approval to look at older specimens Therefore, lowering the concentration of Shh signalling, either endogenously or ectopically, has the same effect on *Sim1* expression (compare Fig. 5F with A).

These findings reveal that Shh signalling from the polarising region between HH18 and HH22 specifies the later pattern of *Sim1* expression in a defined spatial and temporal sequence, which can be replicated by polarising region grafts made to the anterior margin of the wing bud in a dose-dependent manner. Thus, in reference to the classical positional information of digit patterning (reviewed by Tickle and Towers, 2017), a short exposure of Shh signalling (equivalent to a low concentration) is sufficient for weak expression of *Sim1* in digit 1, and progressively longer exposures (higher concentrations) for expression in the distal part of digit 2, the ulna and then digit 3.

### *Sim1*-expressing cells and flight feather buds are adjacent to the polarising region lineage

**Fig. 6.**
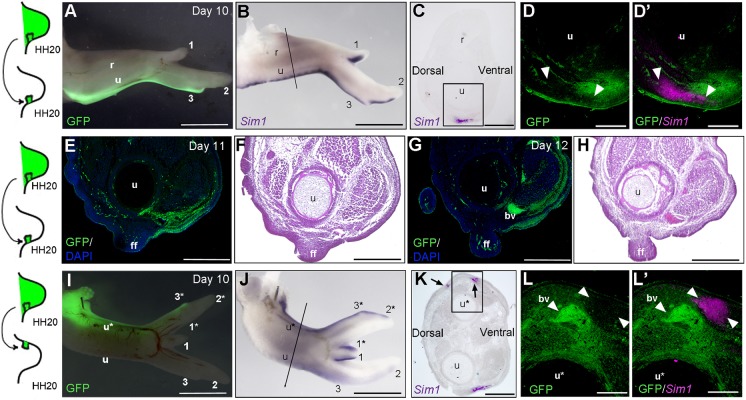
***Sim1* and flight feather buds are adjacent to the polarising region lineage.** (A) GFP-expressing polarising regions transplanted in place of normal polarising regions at HH20 contribute to posterior soft tissues of day 10 wings (green labelling, *n*=2/2; r, radius; u, ulna; 1-3, digits 1, 2 and 3). (B,C) *Sim1* expression along posterior margin of the ulna and digit 3 (*n*=2/2; the same limb as shown in A). (D,D′) Transverse section through forewing shown in A and B reveals adjacent expression of GFP and *Sim1* in dermis (*n*=2/2; GFP protein and *Sim1* mRNA are detected on same section). Arrowheads indicate the domain of *Sim1* expression. (E,G) Transverse sections through a day 11 wing (E, *n*=2/2, experiment performed as in A) and a day 12 wing (G, *n*=2/2) showing GFP expression ventral to emerging flight feather (ff; bv, blood vessel; u, ulna; blue shows DAPI staining). (F,H) Hematoxylin and Eosin staining on serial sections to those in E,G show tissue anatomy. (I) HH20 GFP-expressing polarising region grafts made to the anterior margin of a host wing bud duplicate the distal structures of day 10 wings (*n*=5/5). (J,K) GFP-expressing polarising region grafts duplicate the pattern of *Sim1* expression (*n*=5/5; there is a second line of *Sim1* expression, *n*=3/5; this is the same limb as in I). (L,L′) *Sim1* expression is found adjacent to the polarising region lineage (*n*=5/5). Unlabelled arrowheads indicate the domain of *Sim1* expression. Asterisks indicate duplicated skeletal elements. Scale bars: 1 mm in A,B,I,J; 150 μm in E-H; 75 μm in C,D,K,L.

The pattern of *Sim1* expression along the posterior margin of the wing superficially resembles polarising region fate maps (Towers et al., 2011). Therefore, to examine this putative lineage relationship, we replaced normal HH20 polarising regions with stage-matched GFP-expressing polarising regions and analysed the expression of *Sim1* at day 10 (Fig. 6A,B). Transverse sections show polarising region-derived GFP-expressing dermal cells lying immediately ventral to both *Sim1*-expressing cells in forewings at day 10 (Fig. 6C,D). In addition, Hematoxylin and Eosin staining also shows that GFP-expressing dermal cells abut emerging flight feathers at days 11 and 12 (Fig. 6E-H). These findings show that cells positioned dorsally adjacent to the polarising region express *Sim1* and produce flight feather buds. In addition, the GFP-expressing polarising region lineage and *Sim1*-expressing cells also abut one another in anterior regions of mirror image-duplicated wings (Fig. 6I-L′). We often observed a second stripe of *Sim1* expression along the anterior margin of such wings, but the significance of this is unclear (Fig. 6J,K). Taken together, these findings imply that paracrine Shh signalling induces *Sim1* expression and flight feather formation in cells immediately dorsal to the polarising region lineage.

### Shh signalling is required for flight feather formation in hatchlings

**Fig. 7.**
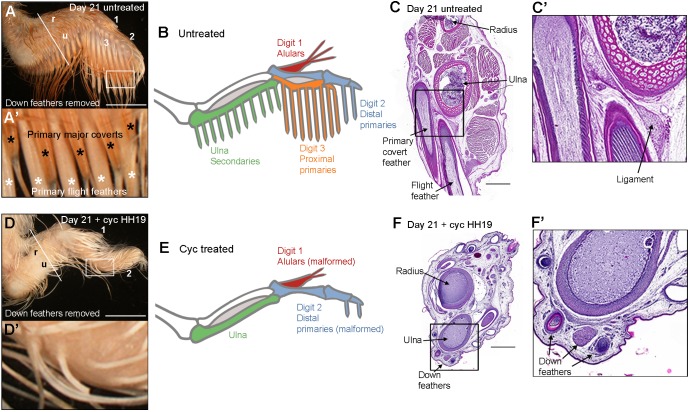
**Shh signalling is required for flight feather formation in the wings of hatchlings.** (A) An example of an untreated chicken wing at hatching (incubation day 21) showing the normal pattern of primary flight feathers and primary major covert feathers (*n*=10/10). (A′) Enlarged area of the wing shown in A. (B) Schematic of normal flight feather pattern at hatching. (C) Hematoxylin and Eosin staining on a transverse section through the wing in A showing ligaments connecting the flight feathers to the ulna. (C′) Enlarged area of section shown in C. (D) Example of a HH19 cyclopamine-treated wing at hatching showing loss of both primary flight feathers and primary major covert feathers (*n*=13/16). (D′) Enlarged area of the image shown in D. (E) Schematic of flight feather pattern in the cyclopamine-treated wing at hatching. Malformed flight feathers often form in distal regions, but can only be seen when natal down is fully removed (see Fig. S4 for examples). (F) Hematoxylin and Eosin staining on a transverse section through the wing in D shows that down feathers are still present at the posterior margin of the wing where flight feathers would normally develop. (F′) Enlarged part of section shown in F. Scale bars: 8 mm in A,D; 1 mm in C,F.

During the stages leading up to hatching (days 18-21 of incubation), the first generation of flight feathers is replaced by the second generation of mature flight feathers (Lucas and Stettenheim, 1972; Kondo et al., 2018). To determine whether polarising region-derived Shh signalling is required for the formation of mature flight feathers, we obtained licensing and ethical permission to allow chicks treated with cyclopamine at HH19 to hatch on day 21 of incubation. In untreated wings, both well-formed flight feathers and dorsal major covert feathers can be observed extending from the posterior margin of the wing (Fig. 7A,A′, most of the natal down is trimmed back). A schematic documenting the association between the flight feathers and the digit skeleton at hatching is shown in Fig. 7B. Hematoxylin and Eosin staining on a transverse section through the forewing region of the specimen in Fig. 7A shows a ligament connecting the flight feather to the ulna (Fig. 7C,C′).

In hatchlings that were treated with cyclopamine at HH19, most flight feathers and dorsal major covert feathers fail to develop at hatching (Fig. 7D,D′, natal down trimmed back, Table S1). A schematic of this pattern of flight feathers with the other feathers completely removed is shown in Fig. 7E [some stunted alulars and secondaries are present but obscured by natal down (see  Fig. S4 and Table S1)]. These are the exact locations where restricted expression of *Sim1* is often observed following the inhibition of Shh signalling at HH19 (Fig. 5B). Hematoxylin and Eosin staining of a section from the wing in Fig. 7D shows that natal down feathers still form in areas in which flight feathers are normally present and ligaments connecting feathers to the ulna are not found (Fig. 7F,F′). In addition, proximal primaries along the border of digit 3 are preferentially lost following the application of cyclopamine at HH21 (Table S1); this also matches the loss of *Sim1* expression in this domain (Fig. 5C). Furthermore, the flight feather pattern is usually normal following cyclopamine treatment at HH22 (Table S1), just as the *Sim1* expression pattern is normal (Fig. 5D). Therefore, Shh signalling by the polarising region specifies the spatial pattern of *Sim1* expression and flight feather formation in the same temporal sequence.

### Shh signalling is required for flight feather formation in mature birds

Hatchlings that were treated with cyclopamine at HH19 did not survive owing to the failure to close the abdominal wall and this prevented the study of their mature wing plumage. As our analyses of hatched chickens shows that dorsal major coverts – which are closely associated with developing flight feathers – are also absent, this raised the possibility that Shh inhibition could affect the later development of other feathers that had not yet replaced the natal down.

**Fig. 8.**
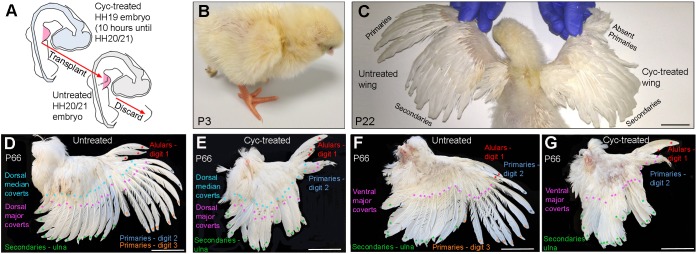
**Shh signalling is required for flight feather formation in the wings of mature birds.** (A) Experimental procedure in which an embryo was treated with cyclopamine at HH19 for 10 h and then its right-hand wing was grafted in place of a stage-matched wing bud of an untreated embryo at HH20/21. (B) Example of a hatched chick (p3, postnatal day 3) that underwent the procedure described in A (see Table S2 for details of the other five chickens that hatched). (C) Same chicken as shown in B at p22. Primary flight feathers are absent in the distal regions of the cyclopamine-treated wing. (D,E) Dorsal views of untreated (D) and cyclopamine-treated (E) wings at p66 (same chicken as in B,C) showing alular flight feathers (red asterisks, digit 1), distal primary flight feathers (blue asterisks, digit 2), proximal primary flight feathers (orange asterisks, digit 3), secondary flight feathers (green asterisks, ulna), dorsal major covert feathers (purple asterisks) and dorsal median covert feathers (light-blue asterisks). Proximal primary flight feathers and overlying primary major covert feathers are absent in the cyclopamine-treated wing (E). (F,G) Ventral views of untreated (F) and cyclopamine-treated (G) wings at p66 showing alular flight feathers (red asterisks, digit 1), distal primary flight feathers (blue asterisks, digit 2), proximal primary flight feathers (orange asterisks, digit 3), secondary flight feathers (green asterisks, ulna) and ventral major covert feathers (purple asterisks). Proximal primary flight feathers are absent in the cyclopamine-treated wing (G). Scale bars: 3 cm in C; 5 cm in D-G.

To analyse the second generation of feather development in the wings of mature birds, we treated embryos at HH19 with cyclopamine, and then after 10 h at HH20/21, we grafted their right-hand wing buds in place of those of stage-matched untreated embryos (Fig. 8A; see control experiment showing that the grafting procedure does not affect mature postnatal feather development in Fig. S5). This procedure enabled six chicks to survive beyond hatching (Fig. 8B) and they displayed similar patterns of flight feather loss as hatched chicks that were systemically treated with cyclopamine as embryos (Tables S1 and S2). Three birds survived further and their patterns of flight feather loss remained the same as at hatching (Tables S1 and S2). One such example of a postnatal day 22 bird shows that the flight feather pattern is normal in its untreated left-hand wing, but that there is a loss of distal primary flight feathers in its cyclopamine-treated right-hand wing (Fig. 8C). This bird was kept until postnatal day 66, so that its adult feather pattern could be studied in more detail (Fig. 8D-G): 18 secondary flight feathers develop from the ulnar region of its control left wing (green asterisks, Fig. 8D,F) and ten primary feathers from its digital region [eight primaries from digit 3, orange asterisks; two from digit 2, blue asterisks, Fig. 8D,F (one feather was broken)]. Three alular flight feathers extending from digit 1 are also present (red asterisks in Fig. 8D,F). However, in the contralateral cyclopamine-treated wing of this bird, eight primary flight feathers and their overlying dorsal major coverts are absent along the posterior margin of digit 3 (Fig. 8E,G). In addition, two bunched primary flight feathers, which are much smaller than the equivalent ones in its control wing, extend from the margin of digit 2 at the distal tip of the wing (blue asterisks, Fig. 8E,G), and overlying them are dorsal major covert feathers (purple asterisks, Fig. 8E). The development of ventral major covert feathers is unaffected by cyclopamine treatment (Fig. 8G). The pattern of feather loss in the wing of this bird is consistent with the pattern of *Sim1* expression in the wings of embryos that were treated with cyclopamine at HH21 (Fig. 5C). It is unclear why we observed stronger effects in cyclopamine-treated embryos at HH19 versus cyclopamine-treated wing buds at HH19 that were transplanted, but it could be a consequence of variability with the effects of cyclopamine treatment. Indeed, the two birds with transplanted wings that survived the longest had the milder flight feather defects (Table S2). These results demonstrate that Shh signalling in the embryo is required for the specification and formation of mature flight feathers, and also their overlying dorsal major coverts in a defined spatial and temporal sequence.

## DISCUSSION

### Embryonic Shh signalling is required for flight feather formation

We have revealed that Shh signalling by the embryonic chick wing polarising region is required for specifying the adult pattern of flight feathers in dorsally adjacent cells. This process is independent of the later role that Shh signalling fulfils in feather morphogenesis (Harris et al., 2002, 2005; McKinnell et al., 2004). Thus, the transient loss of Shh signalling between HH18 and HH22, but not later, causes the loss of bilaterally asymmetric flight feathers that make ligamentous connections to the skeleton, and also the loss of molecular markers associated with the flight feather forming regions of wing. The Shh signalling pathway (*Ptch1* read-out) is still active during later feather bud morphogenesis, demonstrating that this general process is unaffected.

Detailed fate-mapping experiments have shown that when Shh signalling by the polarising region is transiently blocked, the lateral plate mesoderm-derived cells of the early chick wing bud are not selectively lost, but instead contribute to the development of distal structures (Towers et al., 2011, 2008). Therefore, although digit 3 often fails to form, this is a consequence of its progenitor cells being anteriorised and differentiating into digit 2. The fact that dermal tissue is also derived from the lateral plate mesoderm (Pearse et al., 2007), suggests that, when Shh signalling is inhibited in the early wing bud, this induces the formation of feather types in the overlying ectoderm that are usually found in more-anterior positions (i.e. dermal cells which would normally specify flight feathers instead specify major or median coverts).

We have also shown that the dorsal ventral boundary, which is important for flight feather development (Grieshammer et al., 1996), remains intact following the inhibition of Shh signalling. Together, our data provide molecular insights into classical tissue recombination experiments performed in the chick, which show that feather position and identity are specified by signals acting at around HH20 (Cairns and Saunders, 1954; Saunders and Gasseling, 1959).

### Shh specifies flight feather positional information

**Fig. 9.**
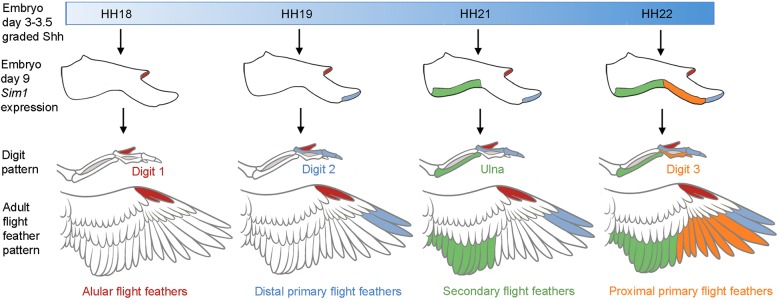
**Positional information model of flight feather specification.** Predicted temporal gradient of Shh from the polarising region between HH18 and HH22 (blue shading, day 3-3.5) is interpreted into a spatiotemporal pattern of *Sim1* expression in flight feather-forming regions of the wing at day 9. The order in which the pattern of flight feathers is specified across the antero-posterior axis is the same as the skeletal elements: alulars, digit 1 (red); distal primaries, digit 2 (blue); secondaries, ulna (green); proximal primaries, digit 3 (orange). The first generation of flight feathers form during late embryogenesis (not shown) and the second generation of flight feathers can be seen in mature wings. The experiments in which embryos were systemically treated with cyclopamine were used to define the temporal requirement of Shh signalling for *Sim1* expression and flight feather development. In addition, chicks treated at HH18 failed to hatch; thus, we predict that alulars would be specified at this stage based on *Sim1* expression.

Our findings can be explained by the classical positional information model of antero-posterior patterning, in which Shh signalling specifies limb bud cells derived from the lateral plate mesoderm, including the presumptive dermis, with a positional value, which when interpreted at a later stage of development allows them to differentiate into the appropriate structure (Tickle et al., 1975; Tickle and Towers, 2017). Thus, the temporal requirement for Shh signalling in specifying the anterior to posterior pattern of *Sim1* expression and flight feathers closely follows that for specifying the anterior to posterior pattern of digits (Yang et al., 1997; Towers et al., 2011; Tickle and Towers, 2017) (Fig. 9). Digit 1 and alular flight feathers are specified first by a low concentration/short duration of Shh at HH18, and then increasing concentrations of Shh over time specify the other skeletal elements and flight feathers in the following order: digit 2 and distal primaries at HH19, the ulna and secondaries at HH21, and digit 3 and proximal primaries at HH22 (Fig. 9). It is noteworthy that flight feather specification closely follows ulna/digit specification, which can be observed by normally patterned skeletal elements forming without associated flight feathers, but with feathers of different identities (Fig. 9). This indicates that a slightly longer exposure to Shh signalling is required for the specification of flight feathers, relative to their associated skeletal elements.

The pattern of *Sim1* expression in the wings of embryos treated at HH19, closely matches the pattern of flight feathers present both at hatching and in mature bird wings (Fig. 9). In the treated wing of the oldest bird we studied (P66) eight proximal primaries – which normally form along the border of digit 3 – are absent, yet two distal primaries are present along the border of digit 2 (Fig. 9). This pattern of flight feather loss is also accompanied by the loss of an associated row of overlying dorsal major covert feathers. However, the inhibition of Shh signalling does not affect the development of the remaining feathers in the wing, including the ventral major coverts, implying that their identities are specified by other signals. Our findings therefore indicate that flight feathers and dorsal major covert feathers have similar developmental programmes. Interestingly, a genetic programme for flight feather development was indicated by naturally occurring mutants that specifically failed to form this feather type (McCrady, 1932; Urrutia et al., 1983). Taken together, these observations suggest that the evolution of the flight feather programme involved the co-option of the pre-existing temporal positional information gradient of Shh signalling used in antero-posterior forewing/digit patterning (Fig. 9).

The interpretation of positional information, in which cells ‘memorise’ their positional value to give rise to appropriately patterned and positioned structures at a later stage of development, is generally a ‘black box’ in developmental biology (Wolpert, 2016). Indeed, despite decades of research, the genes acting downstream of the positional information gradient of Shh signalling in the specification of digit identity remain unknown (Tickle and Towers, 2017). However, our RNA sequencing experiments provide molecular insights into a putative gene regulatory network that operates downstream of Shh signalling in determining flight feather identity. These genes include *Sim1* and, notably, genes encoding three Zic transcription factors (*Zic1*, *Zic3* and *Zic4*). Interestingly, Zic transcription factors can bind to sites in promoters that are also recognised by the Gli family of transcription factors – the downstream effectors of Shh signalling (Aruga et al., 1994). This mechanism could involve positional memory, in which polarising region-derived Shh signalling could remove Gli transcriptional repressors from the regulatory elements of genes, thus making them accessible to Zic transcription factors at stages of flight feather bud development. Such directions could be the focus of future studies. In conclusion, as flight feathers were one of the earliest known adaptations associated with the evolution of flight in theropod dinosaurs (Turner et al., 2007; Ortega et al., 2010; Godefroit et al., 2013), our findings have significant implications for this extraordinary transition.

## MATERIALS AND METHODS

### Chick husbandry

Wild-type and GFP-expressing Bovans brown chicken eggs were incubated and staged according to the Hamburger Hamilton staging table (Hamburger and Hamilton, 1951). Day 3 of incubation is HH18, day 4 is HH21, day 5 is HH24, day 6 is HH27, day 7 is HH29, day 8 is HH30, day 10 is HH36, day 11 is HH37, day 12 is HH38, day 13 is HH39, day 14 is HH40, day 15 is HH41 and hatching (at day 21) is HH46. All experiments involving the use of hatching chicken embryos in this work were conducted in accordance with the EU animal experiment guidelines and reviewed and approved by the Bioethics Committee of the University of Cantabria (PI-20-17).

### Wing bud and polarising region grafts

Embryos were dissected in DMEM and wing buds removed using fine tungsten needles, grafted in place of stage-matched host limb buds and held in place with 25 μm platinum pins. Polarising regions were grafted to the anterior and posterior margins of the wing buds of host embryos (tissue sizes 100 μm^3^ for normal grafts, 25 μm^3^ for small grafts) and held in place with 25 μm platinum pins (Stainton and Towers, 2018).

### Hematoxylin and Eosin staining

Transverse sections (12 μm) of paraffin embedded forewings were mounted on glass slides. Slides were washed twice in xylene for 5 min followed by rehydration through an ethanol series (2×100%, 95% and 70%), and washed in H_2_O. Slides were stained for 2 min in Harris Haematoxylin followed by differentiation in 0.3% acid alcohol. Blueing was achieved in Scott's tap water and slides were rinsed in H_2_O before staining in Eosin for 5 min. Slides were rinsed in H_2_O and dehydrated through an ethanol series (70%, 95% and 100%). Dehydrated slides were cleared of remaining wax with xylene before mounting.

### Shh signalling inhibition

Cyclopamine (Sigma) was suspended in a carrier (45% 2-hydropropyl-β-cyclodextrin in PBS, Sigma, to a concentration of 1 mg/ml). 4 μl was pipetted directly onto embryos over the limb bud, after removal of vitelline membranes. In all cases, untreated wings were treated with 2-hydropropyl-β-cyclodextrin only. Digit identities were determined by visualising phalanges under illumination.

### Whole-mount RNA *in situ* hybridisation

Embryos were fixed in 4% PFA overnight at 4°C, dehydrated in methanol overnight at −20°C, rehydrated through a methanol/PBS series, washed in PBS, then treated with proteinase K for 20 min (10 μg/ml^−1^), washed in PBS, fixed for 30 min in 4% PFA at room temperature and then pre-hybridised at 65°C for 2 h (50% formamide/50% 2× SSC). Antisense DIG-labelled (Roche) mRNA probes (1 μg) were added in 1 ml of hybridisation buffer (50% formamide/50% 2× SSC) at 65°C overnight. Embryos were washed twice in hybridisation buffer, twice in 50:50 hybridisation buffer:MAB buffer and then twice in MAB buffer, before being transferred to blocking buffer (2% blocking reagent 20% lamb serum in MAB buffer) for 2 h at room temperature. Embryos were transferred to blocking buffer containing anti-digoxigenin antibody (Roche 1:2000) at 4°C overnight, then washed in MAB buffer overnight before being transferred to NTM buffer containing NBT/BCIP and mRNA distribution visualised using a LeicaMZ16F microscope.

### Double RNA *in situ* hybridisation/immunohistochemistry

Whole-mount RNA *in situ* hybridisation was performed as above. Embryos were fixed for 20 min at room temperature in 4% PFA, washed twice in PBT for 10 min and then dehydrated through an ethanol series (10 min each wash in PBT) to 100% ethanol and stored at −20°C overnight. Embryos were cleared in xylene until light was visible through the tissue (∼2-10 min). Embryos were processed through a series of 30 min wax changes at 60°C (25%, 25%, 50%, 75%, 100% and 100%) and then left in the oven overnight. Limbs were embedded in wax and allowed to set for 4-6 h before being sectioned using a microtome. Sections were floated on a slide rack overnight at 52°C. Slides were washed in xylene for 5 min (2×) in a Coplin jar, rehydrated through an ethanol series (2×5 min washes each) to H_2_O and then washed twice in PBT. Slides were blocked horizontally for 1 h in 3% HINGS in PBT and incubated in primary antibody (anti-chick GFP at 1:100) in blocking solution overnight at 4°C or for 4 h at room temperature. Slides were washed in a Coplin jar (three times for 15-30 min) and then incubated with secondary antibody goat anti-chicken conjugated to Alexa 488 at 1:500) in blocking solution in the dark. Slides were rinsed four or five times in the dark in PBS and mounted with Fluoroshield (with DAPI).

### RNA sequencing analyses and clustering

Tissue used for making RNA was manually dissected using fine forceps. Three replicate experiments were performed from each condition and the tissue from several embryos was pooled before the RNA was extracted using Trizol reagent (Gibco). Sequencing libraries were prepared using Illumina TruSeq library preparation kit. Samples were sequenced on a HiSeq 2000 (Paired end readings of 50 bp - Instrument: ST300). Reads were aligned to the chicken genome, assembly Gallus_gallus-5.0, using STAR aligner.

The raw RNA sequencing data have been deposited in array express under accession number E-MTAB-7520. A total of 12 samples (three replicates for each condition) were QC analysed using automatic outlier detection. This was carried out by manually inspecting the density plot, boxplots, PCA plots, correlation heatmap and distance plot, as well as using several automatic outlier tests, i.e. distance, Kolmogorov–Smirnov, correlation and Hoeffding's D (all samples passed QC). The read-count data for the samples were normalised using trimmed mean of M-values normalisation and transformed with Voom, resulting in counts per million with associated precision weights. Negative values result when expression is between zero and one before log_2_ transformation. Genes were clustered using the clValid R package based on their log_2_ fold changes. The Dunn Index was selected as the preferred cluster validation measure. Three clustering methods (hierarchical, k-means and PAM) were tested for up to 20 clusters and the clustering analysis was performed on 906 unique genes that were differentially expressed in these contrasts at the significance threshold of FDR-adjusted *P*-value <0.005 and fold change ≥2. Using k-means, the clustering of the 906 genes resulted in four groups.

### qPCR analysis

cDNA was prepared from RNA that was used in the sequencing analysis using SuperScript II Reverse Transcriptase (Invitrogen). qPCR was performed on an Applied Biosystems StepOne RT-PCR machine using TaqMan Fast Advanced Master Mix (Thermo Fisher Scientific) and a TaqMan probe and primer set designed against chicken *Sim1* (Thermo Fisher Scientific). 5 ng cDNA was used per reaction (20 μl volume) with cycle conditions of 95°C for 20 s, followed by 32 cycles of 95°C for 1 s and 60°C for 20 s. All reactions were carried out in triplicate and normalized against eukaryotic *18S rRNA* expression (Thermo Fisher Scientific). Standard errors of the means were generated from the triplicate C_T_ values. Unpaired *t*-tests measured significance of expression change between appropriate samples. Applied Biosystems StepOne Software V2.3 was used to analyse the data and generate gene expression comparisons.

## Supplementary Material

Supplementary information

Reviewer comments

## References

[DEV188821C1] Aruga J., Yokota N., Hashimoto M., Furuichi T., Fukuda M. and Mikoshiba K. (1994). A novel zinc finger protein, zic, is involved in neurogenesis, especially in the cell lineage of cerebellar granule cells. *J. Neurochem.* 63, 1880-1890. 10.1046/j.1471-4159.1994.63051880.x 7931345

[DEV188821C2] Cairns J. M. and Saunders J. R. (1954). The influence of embryonic mesoderm on the regional specification of epidermal derivatives in the chick. *J. Exp. Zool.* 127, 221-248. 10.1002/jez.1401270203

[DEV188821C3] Chang W. L., Wu H., Chiu Y. K., Wang S., Jiang T. X., Luo Z. L., Lin Y. C., Li A., Hsu J. T., Huang H. L. et al. (2019). The making of a flight feather: bio-architectural principles and adaptation. *Cell* 179, 1409-1423.e1417. 10.1016/j.cell.2019.11.008 31778655 PMC6953487

[DEV188821C4] Chen C. F., Foley J., Tang P. C., Li A., Jiang T. X., Wu P., Widelitz R. B. and Chuong C. M. (2015). Development, regeneration, and evolution of feathers. *Annu. Rev. Anim. Biosci.* 3, 169-195. 10.1146/annurev-animal-022513-114127 25387232 PMC5662002

[DEV188821C5] Coumailleau P. and Duprez D. (2009). Sim1 and Sim2 expression during chick and mouse limb development. *Int. J. Dev. Biol.* 53, 149-157. 10.1387/ijdb.082659pc 19123137

[DEV188821C6] Godefroit P., Demuynck H., Dyke G., Hu D., Escuillie F. and Claeys P. (2013). Reduced plumage and flight ability of a new Jurassic paravian theropod from China. *Nat. Commun.* 4, 1394 10.1038/ncomms2389 23340434

[DEV188821C7] Grieshammer U., Minowada G., Pisenti J. M., Abbott U. K. and Martin G. R. (1996). The chick limbless mutation causes abnormalities in limb bud dorsal-ventral patterning: implications for the mechanism of apical ridge formation. *Development* 122, 3851-3861. 9012506 10.1242/dev.122.12.3851

[DEV188821C8] Hamburger V. and Hamilton H. L. (1951). A series of normal stages in the development of the chick embryo. 1951. *J. Morphol.* 88, 49-92. 10.1002/jmor.1050880104 24539719

[DEV188821C9] Harris M. P., Fallon J. F. and Prum R. O. (2002). Shh-Bmp2 signaling module and the evolutionary origin and diversification of feathers. *J. Exp. Zool.* 294, 160-176. 10.1002/jez.10157 12210117

[DEV188821C10] Harris M. P., Williamson S., Fallon J. F., Meinhardt H. and Prum R. O. (2005). Molecular evidence for an activator-inhibitor mechanism in development of embryonic feather branching. *Proc. Natl. Acad. Sci. USA* 102, 11734-11739. 10.1073/pnas.0500781102 16087884 PMC1187956

[DEV188821C11] Kondo M., Sekine T., Miyakoshi T., Kitajima K., Egawa S., Seki R., Abe G. and Tamura K. (2018). Flight feather development: its early specialization during embryogenesis. *Zoological Lett.* 4, 2 10.1186/s40851-017-0085-4 29372073 PMC5771061

[DEV188821C12] Lewis J. H. and Wolpert L. (1976). The principle of non-equivalence in development. *J. Theor. Biol.* 62, 479-490. 10.1016/0022-5193(76)90132-6 1086930

[DEV188821C13] Lucas A. M. and Stettenheim P. R. (1972). Avian anatomy - integument. *Agriculture handbook* 362, 280-308.

[DEV188821C14] Luxey M., Berki B., Heusermann W., Fischer S. and Tschopp P. (2020). Development of the chick wing and leg neuromuscular systems and their plasticity in response to changes in digit numbers. *Dev. Biol.* 458, 133-140. 10.1016/j.ydbio.2019.10.035 31697937

[DEV188821C15] Matloff L. Y., Chang E., Feo T., Jeffries L., Stowers A., Thomson C. and Lentink D. (2020). How flight feathers stick together to form a continuous morphing wing. *Science* 367, 293-297. 10.1126/science.aaz3358 31949079

[DEV188821C38] McCrady E. (1932). The ‘penguin’ guinea fowl: absence of flight feathers due to hereditary local alopecia. *J. Hered.* 23, 201-207.

[DEV188821C16] McKinnell I. W., Turmaine M. and Patel K. (2004). Sonic Hedgehog functions by localizing the region of proliferation in early developing feather buds. *Dev. Biol.* 272, 76-88. 10.1016/j.ydbio.2004.04.019 15242792

[DEV188821C17] Michon F., Forest L., Collomb E., Demongeot J. and Dhouailly D. (2008). BMP2 and BMP7 play antagonistic roles in feather induction. *Development* 135, 2797-2805. 10.1242/dev.018341 18635609 PMC2659359

[DEV188821C18] Ortega F., Escaso F. and Sanz J. L. (2010). A bizarre, humped Carcharodontosauria (Theropoda) from the lower cretaceous of Spain. *Nature* 467, 203-206. 10.1038/nature09181 20829793

[DEV188821C19] Pearse R. V. II, Scherz P. J., Campbell J. K. and Tabin C. J. (2007). A cellular lineage analysis of the chick limb bud. *Dev. Biol.* 310, 388-400. 10.1016/j.ydbio.2007.08.002 17888899 PMC2940718

[DEV188821C20] Pickering J. and Towers M. (2016). Inhibition of Shh signalling in the chick wing gives insights into digit patterning and evolution. *Development* 143, 3514-3521. 10.1242/dev.137398 27702785 PMC5087615

[DEV188821C21] Prin P. and Dhouailly D. (2004). How and when the regional competence of chick epidermis is established: feathers vs. scutate and reticulate scales, a problem en route to a solution. *Int. J. Dev. Biol.* 48, 137-148. 10.1387/ijdb.15272378 15272378

[DEV188821C22] Riddle R. D., Johnson R. L., Laufer E. and Tabin C. (1993). Sonic hedgehog mediates the polarizing activity of the ZPA. *Cell* 75, 1401-1416. 10.1016/0092-8674(93)90626-2 8269518

[DEV188821C23] Ros M. A., Dahn R. D., Fernandez-Teran M., Rashka K., Caruccio N. C., Hasso S. M., Bitgood J. J., Lancman J. J. and Fallon J. F. (2003). The chick oligozeugodactyly (ozd) mutant lacks sonic hedgehog function in the limb. *Development* 130, 527-537. 10.1242/dev.00245 12490559

[DEV188821C24] Saunders J. W. Jr. and Gasseling M. T. (1959). Effects of reorienting the wing-bud apex in the chick embryo. *J. Exp. Zool.* 142, 553-569. 10.1002/jez.1401420126 13746798

[DEV188821C25] Scherz P. J., McGlinn E., Nissim S. and Tabin C. J. (2007). Extended exposure to Sonic hedgehog is required for patterning the posterior digits of the vertebrate limb. *Dev. Biol.* 308, 343-354. 10.1016/j.ydbio.2007.05.030 17610861 PMC2100419

[DEV188821C26] Seki R., Li C., Fang Q., Hayashi S., Egawa S., Hu J., Xu L., Pan H., Kondo M., Sato T. et al. (2017). Functional roles of Aves class-specific cis-regulatory elements on macroevolution of bird-specific features. *Nat. Commun.* 8, 14229 10.1038/ncomms14229 28165450 PMC5473641

[DEV188821C27] Stainton H. and Towers M. (2018). Polarizing region tissue grafting in the chick embryo limb bud. *Methods Mol. Biol.* 1863, 143-153. 10.1007/978-1-4939-8772-6_8 30324596

[DEV188821C28] Taipale J., Chen J. K., Cooper M. K., Wang B. L., Mann R. K., Milenkovic L., Scott M. P. and Beachy P. A. (2000). Effects of oncogenic mutations in Smoothened and Patched can be reversed by cyclopamine. *Nature* 406, 1005-1009. 10.1038/35023008 10984056

[DEV188821C29] Tickle C. (1981). The number of polarizing region cells required to specify additional digits in the developing chick wing. *Nature* 289, 295-298. 10.1038/289295a0 7453825

[DEV188821C30] Tickle C. and Towers M. (2017). Sonic hedgehog signaling in limb development. *Front. Cell Dev. Biol.* 5, 14 10.3389/fcell.2017.00014 28293554 PMC5328949

[DEV188821C31] Tickle C., Summerbell D. and Wolpert L. (1975). Positional signalling and specification of digits in chick limb morphogenesis. *Nature* 254, 199-202. 10.1038/254199a0 1113884

[DEV188821C32] Towers M., Mahood R., Yin Y. and Tickle C. (2008). Integration of growth and specification in chick wing digit-patterning. *Nature* 452, 882-886. 10.1038/nature06718 18354396

[DEV188821C33] Towers M., Signolet J., Sherman A., Sang H. and Tickle C. (2011). Insights into bird wing evolution and digit specification from polarizing region fate maps. *Nat. Commun.* 2, 426 10.1038/ncomms1437 21829188

[DEV188821C34] Turner A. H., Makovicky P. J. and Norell M. A. (2007). Feather quill knobs in the dinosaur Velociraptor. *Science* 317, 1721 10.1126/science.1145076 17885130

[DEV188821C40] Urrutia M. S., Crawford R. D. and Classen H. L. (1983). Dysplastic remiges, a genetic abnormality reducing feathering in the domestic fowl. *J. Hered.* 74, 101-104. 10.1093/oxfordjournals.jhered.a109728

[DEV188821C35] Wolpert L. (2016). Positional information and pattern formation. *Curr. Top. Dev. Biol.* 117, 597-608. 10.1016/bs.ctdb.2015.11.008 26970003

[DEV188821C36] Wu P., Yan J., Lai Y. C., Ng C. S., Li A., Jiang X., Elsey R. M., Widelitz R., Bajpai R., Li W. H. et al. (2018). Multiple regulatory modules are required for scale-to-feather conversion. *Mol. Biol. Evol.* 35, 417-430. 10.1093/molbev/msx295 29177513 PMC5850302

[DEV188821C37] Yang Y., Drossopoulou G., Chuang P. T., Duprez D., Marti E., Bumcrot D., Vargesson N., Clarke J., Niswander L., McMahon A. et al. (1997). Relationship between dose, distance and time in Sonic Hedgehog-mediated regulation of anteroposterior polarity in the chick limb. *Development* 124, 4393-4404. 9334287 10.1242/dev.124.21.4393

