# Supplementary material for: Sonic hedgehog specifies flight feather positional information in avian wings
Source: Development. 2020 May 5;147(9):dev188821. doi: 10.1242/dev.188821 (PMC7225127; doi:10.1242/dev.188821)
Supplement: Supplementary information [file develop-147-188821-s1.pdf]

Supplementary Data

Sonic hedgehog specifies flight feather positional information in avian wings

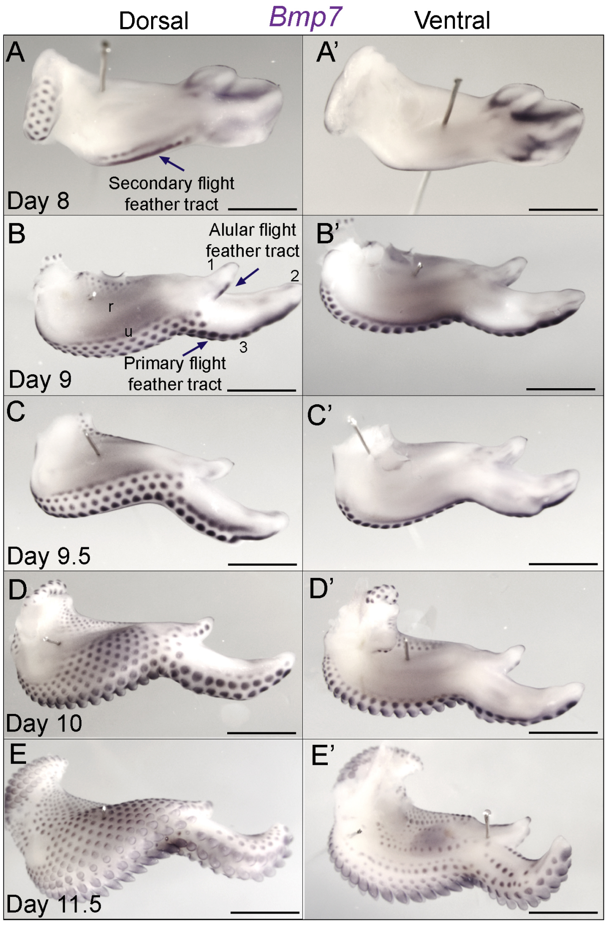

Figure S1—Expression of *Bmp7* during chick wing feather development

(**A-E**) *Bmp7* is expressed in all feather buds. Note, dorsal and ventral views are shown and tracts of feather buds form in a posterior to anterior sequence over time—the first tract to form in the forewing is the secondary flight feather tract at day 8 (A)—the first tract to form in the hand-plate is the primary flight feather tract at day 9 (B).

Scale bars: 1 mm.

Development • Supplementary information

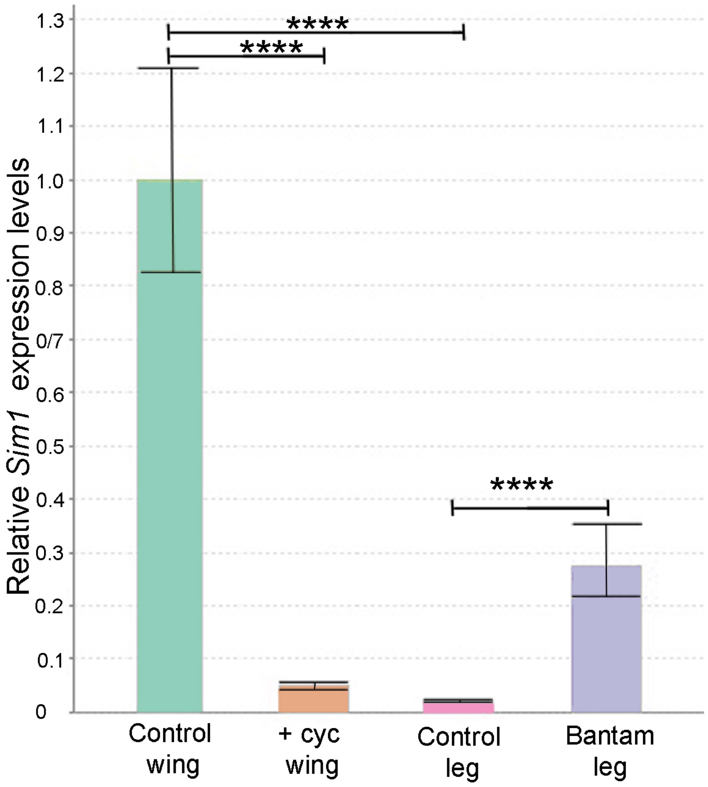

Figure S2—qPCR validation of *Sim1* expression

Sim1 expression is significantly decreased in cyclopamine-treated wing compared to normal wings, and *Sim1* expression is significantly increased in expression both in wings versus legs and in Pekin bantam legs versus Bovans brown legs.

Unpaired *t*-test (**** $p<0.0001$) and error bars indicate standard error.

Development • Supplementary information

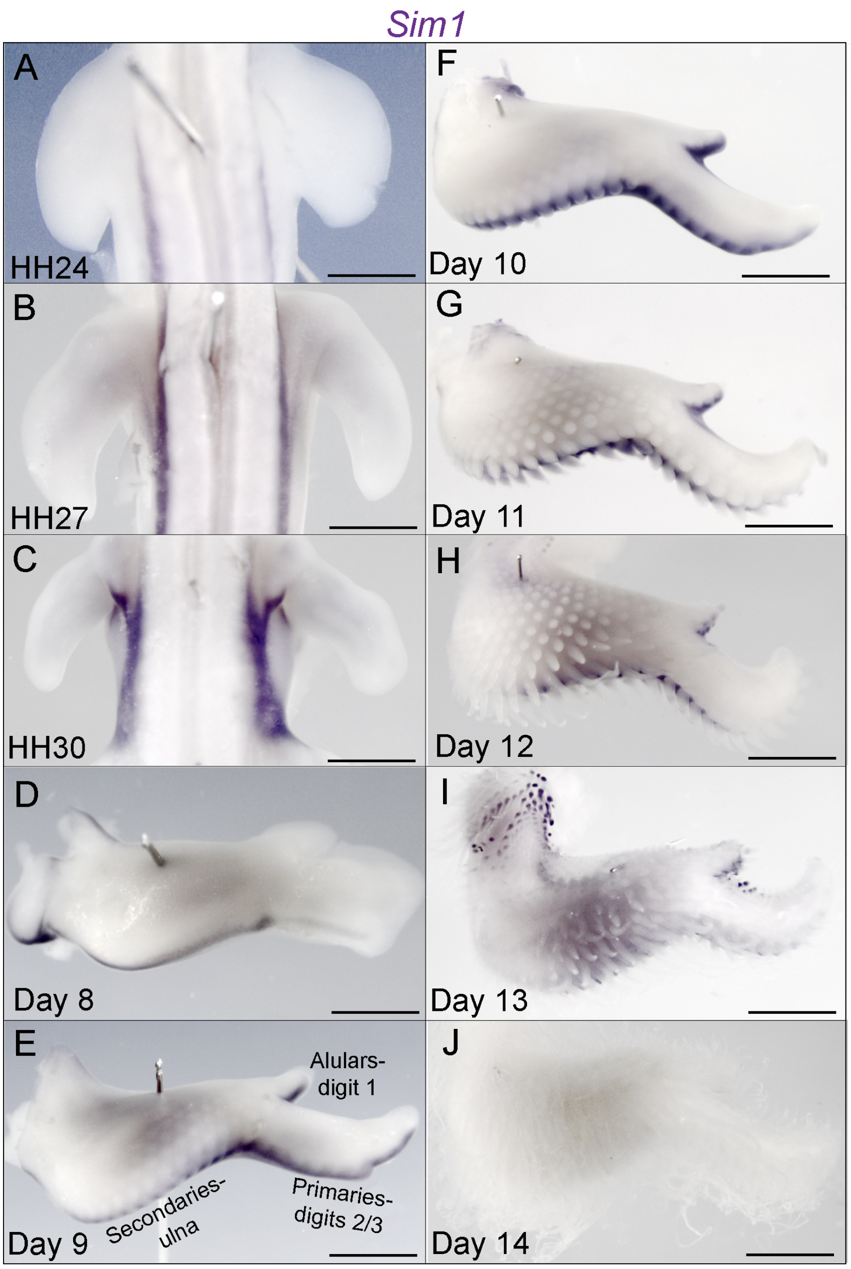

Figure S3—Expression of *Sim1* during chick wing development

A-J) *Sim1* is expressed in flight feather forming regions between day 8 and day 14.

Scale bars: 1 mm.

Development • Supplementary information

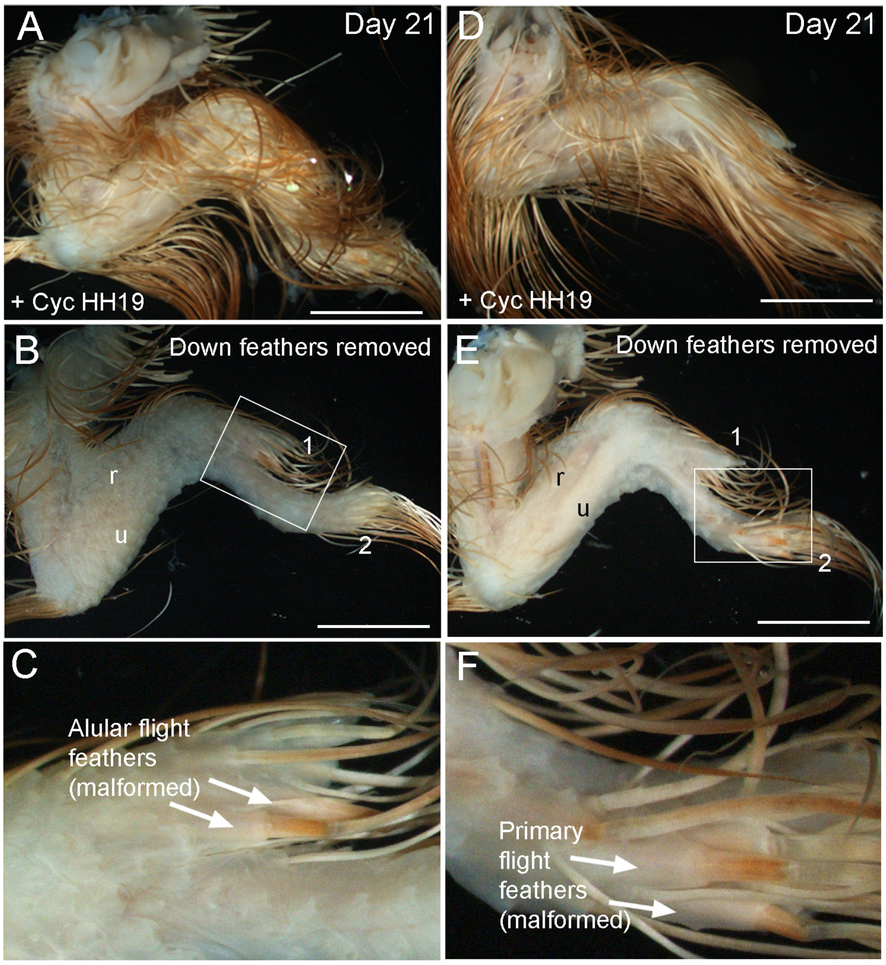

Figure S4—Flight feather development in cyclopamine-treated wings at hatching

A, **D**) Two examples of HH19 cyclopamine-treated wings at hatching in which rudimentary flight feathers can be only observed in distal regions of digit 1 after natal down has been removed (**B**, **C**—alulars, r – radius; u - ulna), and digit 2 (**E**, **F**—distal primaries)—See Supplementary Table 1.

Scale bars: 8 mm.

Development • Supplementary information

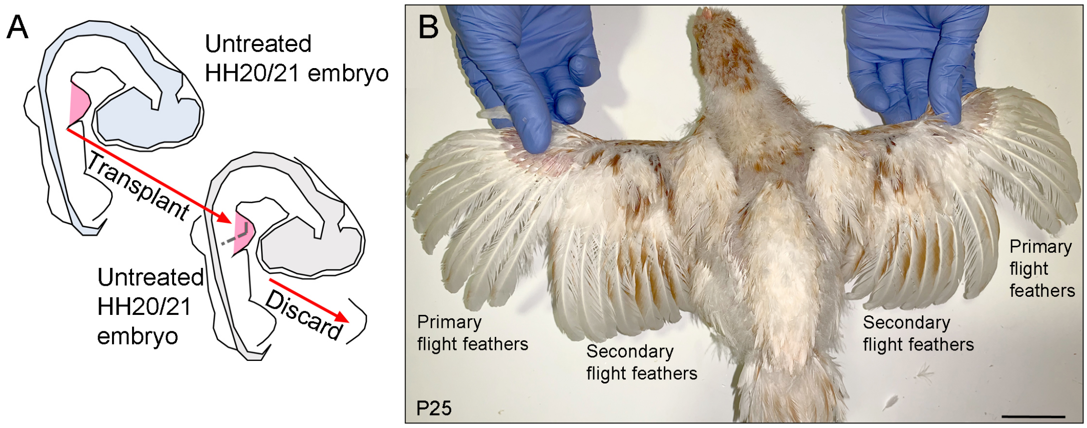

Figure S5—Normal mature feather development in transplanted wings

A) Schematic showing procedure in which an untreated HH20/21 wing bud is grafted in place of a stage-matched wing bud of another embryo. **B**) A chicken that underwent this grafting procedure showing normal development of feathers at p25. Scale bar: 5 cm.

Development • Supplementary information

	Untreated (*n*=10)	Cyc HH19 (*n*=16)	Cyc HH21 (*n*=19)	Cyc HH22 (*n*=3)
Normal flight feathers	10	3	6	2
Scarce underdeveloped alulars and distal primaries. No secondaries	0	11	2	1
Some distal primaries and secondaries No proximal primaries	0	2	11	0

Table S1—Effects of cyclopamine on feather pattern in day 21 chickens at hatching.

	Untreated (*n*=3)	Cyc HH19 (*n*=6)
Normal flight feathers	3	0
Scarce underdeveloped alulars and distal primaries. No secondaries	0	3
Some distal primaries and secondaries No proximal primaries	0	3

Table S2—Effects of cyclopamine applied for 10 hours at HH19 on the mature feather pattern in transplanted wings grafted to untreated host chick embryos at HH20/21. Note out of the six cyc-treated examples, one survived to p37 and one to p66 to allow their full plumage to be studied, and their defects were of the milder category

Table S3. RNA sequencing gene lists

Click here to Download Table S3

Development • Supplementary information
