# Supplementary material for: Sonic hedgehog specifies flight feather positional information in avian wings
Source: Development. 2020 May 5;147(9):dev188821. doi: 10.1242/dev.188821 (PMC7225127; doi:10.1242/dev.188821)
Supplement: Reviewer comments [file develop-147-188821_review_history.pdf]

Sonic hedgehog specifies flight feather positional information in avian wings

Lara Busby, Cristina Aceituno, Caitlin McQueen, Constance A. Rich, Maria A. Ros and Matthew Towers
DOI: 10.1242/dev.188821

Editor: James Briscoe

Review timeline
Original submission: 27 January 2020
Editorial decision: 20 February 2020
First revision received: 4 March 2020
Editorial decision: 23 March 2020
Second revision received: 23 March 2020
Accepted: 24 March 2020

Original submission

First decision letter

MS ID#: DEVELOP/2020/188821

MS TITLE: A positional information gradient of sonic hedgehog specifies flight feather pattern in the avian wing

AUTHORS: Matthew Towers, Lara Busby, Caitlin McQueen, Cristina Aceituno, Constance Rich, and Marian Ros

I have now received all the referees' reports on the above manuscript, and have reached a decision. The referees' comments are appended below, or you can access them online: please go to BenchPress and click on the 'Manuscripts with Decisions' queue in the Author Area.

As you will see, the referees express considerable interest in your work, but have some significant criticisms and recommend a substantial revision of your manuscript before we can consider publication. In the main, I think these constructive comments and addressing them will strengthen and clarify the manuscript. Of the experimental suggestions made by the referees, it seems to me that the transplantation of a ZPA, or Shh expressing cells, to the anterior margin of a limb bud followed by analysis of Sim1 and flight feathers, would be an informative and valuable addition to the study that would tackleseveral of the criticisms.

If you are able to revise the manuscript along the lines suggested, I will be happy receive a revised version of the manuscript. Your revised paper will be re-reviewed by one or more of the original referees, and acceptance of your manuscript will depend on your addressing satisfactorily the reviewers' major concerns. Please also note that Development will normally permit only one round of major revision.

Please attend to all of the reviewers' comments and ensure that you clearly highlight all changes made in the revised manuscript. Please avoid using 'Tracked changes' in Word files as these are lost in PDF conversion. I should be grateful if you would also provide a point-by-point response detailing how you have dealt with the points raised by the reviewers in the 'Response to Reviewers' box. If you do not agree with any of their criticisms or suggestions please explain clearly why this is so.

© 2020. Published by The Company of Biologists under the terms of the Creative Commons Attribution License (https://creativecommons.org/licenses/by/4.0/).

Reviewer 1

Advance summary and potential significance to field

In this manuscript, Busby and colleagues study the role of Shh signaling in the formation of flight feathers in the chick embryo.
Using a series of experiments in which Shh signaling is specifically inhibited using treatments with the cyclopamine alkaloid, authors show that transient, asymmetric expression of Shh at around HH20 is necessary for the formation of flight feather-specific buds in the posterior margin of the wing bud, independently of the formation of posterior structures (since the expression of the Lmx1 marker and the architecture of the wing mesenchyme are normal in treated embryos).
By performing RNA seq of the forming posterior wing region, they pinpoint several genes differentially expressed in the wing bud that may be responsible for flight feather development including previously identified Sim1. Treatments with cyclopamine in a time series and tissue recombination experiments show that pulses of Shh signaling inhibition at different times cause a spatially progressive loss of the Sim1 expression domain, which flanks cells originating from the posterior region (and that have thus expressed Shh at earlier stages).
In an original experimental set up, authors then show that early Shh signaling is also important for the formation of the second wave of flight feathers during embryogenesis (visible in just-hatched individuals), as well as for that of mature flight feathers in adult individuals.
This is a clear, easy-to-read, well-written manuscript. Results are interesting because beyond implications for flight feather development, they address a long standing question, how early signalling mechanisms are integrated and maintained as memory in developing tissues long after source tissues or causal molecules have stopped acting.

Comments for the author

I feel that for publication authors should address the following major comments:

- Cyclopamine is a strong alkaloid; it causes important congenital defects (references should be cited), and while its role in inhibiting Shh signaling has been shown (this should also be referred to and further detailed), its specificity remains questionable. The chick embryo may provide the opportunity to perform complementary functional in which Shh is specifically targeted (e.g., through electroporation of RNA-i carrying constructs in the wing bud).

- Authors choose to test the effect of cyclopamine on the expression of Sim1 or Zic1 previously shown to mark the posterior region of the wing bud. This choice of previously known candidates/markers questions the utility of performing an unbiased RNA seq experiment. If authors wish to maintain mention of this transcriptome analysis, it should be providing insights (i.e., they should show at least the expression pattern of genes identified through this method in treated and un-treated embryos).

Other comments:

- The abstract and introduction mention evolutionary implication of the work. While this is an important –and impactful matter, it remains here purely theoretical (as such, data does not allow making strong evolutionary claims). The manuscript should reflect this, and the rather long evolutionary-oriented part of the introduction would be better suited as a discussion paragraph. Also, please introduce information relative to flight feathers prior to the small summary of results.
- Please provide literature references on the bio-mechanics of flight feathers to support the argument that they support most of flight forces (even though it is a priori intuitive).

- The argumentation is unclear in the first result paragraph: cyclopamine treatment leads to loss of feather buds (and Pitch expression), while no treatment has no effect. It is not possible to conclude from these results that it is the earlier loss of Shh that prevents flight feather formation (a conclusion that can however be made later in the paper). Please clarify.

© 2020. Published by The Company of Biologists under the terms of the Creative Commons Attribution License (https://creativecommons.org/licenses/by/4.0/).

-Please state more clearly the conclusion in the paragraph describing lineage experiments (i.e., the fact that cells expressing Sim1 lie next to cells originating from the PR region shows that they have been exposed to Shh signaling).

-Figure 3: the sequential disappearance of Sim1 expression depending on the timing of cyclopamine administration is one of the strongest experiments of the paper, please present it in a clearer fashion (i.e., provide magnification views in and outside of areas lacking feather buds to clearly show their absence together with that of Sim1 expression)

-Figure 4: Please explain briefly in the text why chicks treated with cyclopamine at HH19 do not survive beyond hatching. Is this expected?

- Figure 5 shows a flight feather phenotype obtained in a cyclopamine-treated, then long-term grafted chick individual. A very long and detailed paragraph is dedicated to this result. It has however (understandably) been obtained in only one individual, and given the artifactual nature of grafting experiments, authors should either repeat the experiment for strong conclusions, or significantly shorten writing / tune-down conclusions.

- It would be nice to see further discussion on the exact role of Shh signaling: does it specify flight feather bud cells, is it necessary for their differentiation (as was previously shown for feather buds in general), or is it required for the spatial patterning of flight feathers?

<u>Reviewer 2</u>

Advance summary and potential significance to field

The manuscript shows that Sonic Hedgehog (Shh) produced by the ZPA at E3 is required for the formation of the first and second generations of adult flight feathers in chicken. They also identified a group of genes differentially expressed in flight feather buds at E10, after cyclopamine exposure at E3 compared to non-treated wings, group of genes that includes Sim1. The presence of Shh at HH18-HH22 is required for Sim1 expression at E10 in posterior margin of the wing. They also show that Sim1 expression pattern at E10 does not overlap but is close to the ZPA-derived cells. Although the involvement of Shh in flight feather formation is somehow expected given the fact that Shh specifies all limb structures across the antero-posterior axis and that flight feathers originate from limb lateral-plate, the authors analyse further the mechanism underlying Shh involvement in flight feather formation.

Comments for the author

One main concern is that text and data do not correlate perfectly. Data appears to be over-interpreted. It is possible that it is just question of writing, I had difficulties to understand the text and did not see data supporting all the conclusions. Here are some exemples.
Summary «Here we show that a positional information gradient of Sonic hedgehog (Shh) signalling in the embryonic chick wing bud specifies the pattern of adult flight feathers in a defined spatial and temporal sequence that reflects their different identities.»
I did not see any data showing this conclusion.
"Figure 3—Shh signalling is interpreted into a spatiotemporal pattern of Sim1 Expression"
The data in Figure 3 do not reflect the title of Figure 3. The data in Figure 3 show that Sim1 expression does not overlap but is close to the ZPA-derived cells. I do not see any data showing an interpretation of the spatiotemporal pattern of Shh.

SIM1 function in flight feather formation
"Sim1 as a marker of flight feather-forming regions" Does it refer to previous lineage tracing experiments that show where are the flight feather-forming regions?
Shh is required for Sim1 expression, but it is not clear what could be the function of Sim1 in flight feather formation downstream of Shh. How does Sim1 expression behave upon different concentrations of Shh applied at the anterior margin? Would Sim1 mutant mice provide any additional information?

© 2020. Published by The Company of Biologists under the terms of the Creative Commons Attribution License (https://creativecommons.org/licenses/by/4.0/).

Additional remarks:
The administration of the Shh inhibitor (cyclopamin) does not target the early limb source of Shh (ZPA), but is delivered in the whole embryo. I was wondering if it was possible to exclude any potential effects of Shh inhibition from other sources (than ZPA) in the embryo.

Introduction page 3-4
Other tissues that are not derived from the lateral plate mesoderm, including the nerves and muscles, are duplicated as a secondary consequence, and thus show equivalence (i.e. are not intrinsically different in character and do not carry positional information).
References are needed for this sentence. A recent one for muscle and innervation could be Luxey et al., 2020 PMID: 31697937.

Results page 5
«The loss of flight feather buds could be interpreted a consequence of missing posterior structures: i.e. digit 3 often does not form in wing buds treated with cyclopamine at HH19»
Does it refer to data in the MS or shall it be referenced?

Figure 1
A schematic of the feather loss upon cyclopamine exposure would be informative.
Figure 4
Here again a schematic of the phenotype would help the reader.

Figure 2 and Figure 3
The panel b of Figure 3 is redundant with the panels f,g of Figure 2. These panels show the same experience: Sim1 expression at E10 after cyclopamine application in early wing buds.

Figure 5 panel h
Graded Shh signalling between HH18 and HH22 (blue shading—h) specifies the spatiotemporal pattern of Sim1 expression and adult flight feathers in the order that the pattern of skeletal elements is also specified across the antero-posterior axis.
I did not find any experimental argument to say this, since there is no experiment with graded Shh signalling.

Material and methods Page 18
"Embryos were dissected in DMEM and wing buds removed using fine tungsten needles, grafted to the appropriate location of stage-matched host limb buds and held in place with 25 µm platinum pins."
« appropriate location » is a bit vague. A more precise explanation would be appreciated.

Reviewer 3

Advance summary and potential significance to field

The manuscript "A positional information gradient of sonic hedgehog specifies flight feather pattern in the avian wing" by Busby et al. examines the consequences of Shh downregulation on flight feather associated gene expression, and on the development of flight feathers. The authors first show that cyclopamine treatment, which inhibits Shh signalling, causes a loss of flight feathers in the chick. This loss is independent of Shh's role in feather morphogenesis and is not a result of a loss of dorsal/ventral patterning. The loss of flight feather buds is reflected in a loss of flight feathers at hatching. The authors then examine gene expression using RNA-seq and show that expression of Sim1 and members of the Zic family are downregulated in response to cyclopamine treatment. This result is verified through in situ hybridisation. The authors go on to show that the extent of downregulation of Sim1 is dependent on the embryonic stage at which Shh is inhibited, and that the spatial pattern of Sim1 and Shh appear correlated. Finally, the authors show that flight feathers are absent in mature (P66) chicks from those regions of the wing which lacked Sim1 expression. Based on these results, the authors conclude that Shh controls the expression of Sim1 in a spatiotemporally integrated manner, ultimately controlling the patterning of flight feathers. They further suggest that flight feather patterning co-opted the previously existing Shh gradient which patterns digits in the forelimb and that this transition was crucial to the evolution of wings. The

© 2020. Published by The Company of Biologists under the terms of the Creative Commons Attribution License (https://creativecommons.org/licenses/by/4.0/).

authors corroborate the previous finding that Sim1 is associated with flight feather development (Seki et al. 2017) and advance current knowledge by showing that expression of this gene is affected by Shh inhibition. The data presented do justify the conclusions drawn to an extent, but additional work to investigate the consequence of ectopic Shh expression be necessary to fully support their conclusions. The experiments presented appear well controlled. The manuscript suffers, however, from mislabelling and some lack of clarity, which means that the reader is sometimes unsure as to the experimental conditions for some of the presented data. The work is interesting, but several revisions are required before the manuscript can be recommended for publication.

Comments for the author

Major Concerns
1) Figure 3 nicely shows the progressive loss of Sim1 expression with earlier treatment by cyclopamine and the correlation between Sim1 expression and transplanted ZPA tissue. Figure 4 and 5 show how this affects the formation of mature flight feathers. However, to determine that Shh is necessary and sufficient for Sim1 expression, and subsequent flight feather development, the authors should show that Shh can induce ectopic Sim1 and flight feathers. This could be done by transplantation of a ZPA from a GFP expressing donor, or cultured Shh expressing cells, to the anterior margin of the limb bud followed by in situ for Sim1, and analysis of mature flight feathers. This would show that Shh is both required for, and capable of inducing, flight feather patterning.

2) The results from the last two experiments seem unclear. Figure 4 appears to show that embryos treated at HH19 form no flight feathers. Supplementary figure 3 is described in the text as showing that later treatment (HH20/21) by cyclopamine leads to formation of flight feathers on distal structures. However, the figure and figure legend imply that the treatment was at HH19. Figure 5 suggests that treatment at HH19 will lead to formation of distal primary flight feathers, in agreement with supplementary figure 3, but disagreeing with figure 4. Additionally, in figure 5, transplantation of a limb from an embryo treated with cyclopamine at HH19/20 (although listed in supplementary data as HH20) should lead to formation of only distal primary flight feathers and alular feathers if the schematic is correct, however all secondary flight feathers appear present. This discrepancy in the data needs to be addressed. Fig 5h implies that the treatments and transplants was carried out at all the stages listed and the results illustrated, however, treatment was at HH20 or 20/21 for all hatched birds. It is not clear that the diagram in Fig 5h is a schematised hypothesis. The authors should clarify how the experiments were done and further provide their clear result for the different experiments.

3) For the RNA-seq experiment, the controls are not clear for Fig. 2e, j, o and t. The y-axis is labelled Log2 fold expression, however, it is not clear against which control the fold change was calculated. The Bovan's brown leg should be used as the control, but its fold change appears to vary. In contrast to the graphs shown, the figure caption describes the y axis as normalised read-count intensities, however at least two graphs show negative values on in the box plot. The authors should make their controls clear and fix the discrepancy between the figure and the figure legend. This experiment would benefit from qPCR validation of the differences in mRNA transcript levels between the 4 conditions.

Minor Concerns
1) The introduction could benefit from a few more citations, and more considered explanation. For example, the statement "Other tissues that are not derived from the lateral plate mesoderm, including the nerves and muscles, are duplicated as a secondary consequence, and thus show equivalence" is uncited and somewhat unclear. Similarly, in the results section, conclusions are drawn based on results from previous papers which are not fully explained. For example, the end of page 5 and beginning of page 6 – it is not clear why the conversion to more anterior structures should have no impact on feather formation.

2) Figure captions are confusing as the panel letter is often listed after the described panel, rather than before. This can make it difficult to understand which panel is being described.

3) Fig 3h and h' are missing scale bars

© 2020. Published by The Company of Biologists under the terms of the Creative Commons Attribution License (https://creativecommons.org/licenses/by/4.0/).

4) Unclosed parentheses at the bottom of page 7

First revision

Author response to reviewers' comments

Reviewer 1 Advance Summary and Potential Significance to Field:
In this manuscript, Busby and colleagues study the role of Shh signaling in the formation of flight feathers in the chick embryo.
Using a series of experiments in which Shh signaling is specifically inhibited using treatments with the cyclopamine alkaloid, authors show that transient, asymmetric expression of Shh at around HH20 is necessary for the formation of flight feather-specific buds in the posterior margin of the wing bud, independently of the formation of posterior structures (since the expression of the Lmx1 marker and the architecture of the wing mesenchyme are normal in treated embryos).
By performing RNA seq of the forming posterior wing region, they pinpoint several genes differentially expressed in the wing bud that may be responsible for flight feather development, including previously identified Sim1. Treatments with cyclopamine in a time series and tissue recombination experiments show that pulses of Shh signaling inhibition at different times cause a spatially progressive loss of the Sim1 expression domain, which flanks cells originating from the posterior region (and that have thus expressed Shh at earlier stages). In an original experimental set up, authors then show that early Shh signaling is also important for the formation of the second wave of flight feathers during embryogenesis (visible in just-hatched individuals), as well as for that of mature flight feathers in adult individuals.
This is a clear, easy-to-read, well-written manuscript. Results are interesting because beyond implications for flight feather development, they address a long standing question, how early signalling mechanisms are integrated and maintained as memory in developing tissues long after source tissues or causal molecules have stopped acting.

Reviewer 1 Comments for the Author:
I feel that for publication authors should address the following major comments:

-Cyclopamine is a strong alkaloid; it causes important congenital defects (references should be cited), and while its role in inhibiting Shh signaling has been shown (this should also be referred to and further detailed), its specificity remains questionable. The chick embryo may provide the opportunity to perform complementary functional in which Shh is specifically targeted (e.g., through electroporation of RNA-i carrying constructs in the wing bud).

We have included references detailing the use of cyclopamine to target the Shh signalling pathway. The effects of cyclopamine in the limb have been documented many times by us and other labs (papers now referenced). All observed effects are consistent with the knockdown of the Shh signalling pathway, including loss of Shh target gene expression, loss of posterior digits and now the flight feathers. In fact, the wing and leg phenotypes obtained by cyclopamine treatment match those found in the chicken *oligozeugodactly* mutant (which unfortunately is unavailable) in which Shh signalling is completely lost specifically in the limb (now referenced- line 117). Therefore, we are confident that cyclopamine is specifically affecting Shh signalling, and we do not think it is worthwhile to use any other methods. In addition, other approaches such as RNAi are not as effective due to the mosaic nature of electroporation and the temporal imprecision inherent to such techniques.

-Authors choose to test the effect of cyclopamine on the expression of Sim1 or Zic1, previously shown to mark the posterior region of the wing bud. This choice of previously known candidates/markers questions the utility of performing an unbiased RNA seq experiment. If authors wish to maintain mention of this transcriptome analysis, it should be providing insights (i.e., they should show at least the expression pattern of genes identified through this method in treated and un-treated embryos).

© 2020. Published by The Company of Biologists under the terms of the Creative Commons Attribution License (https://creativecommons.org/licenses/by/4.0/).

Although Sim1 and Zic1 have been previously identified, our experimental set-up using four conditions provides much more information than was previously available about the regulation/expression of these genes and their implications in feather development. In addition, we did not know that *Sim1* would be the best marker of flight feather development until we performed the analysis. However, the use of *Sim1* was justified by our further analysis showing that Shh signalling temporally controls its later expression in all flight feather-forming regions. We think the data is worthwhile for these reasons, and it also provides a valuable resource for other researchers who may wish to understand the functions of these genes in feather development, both in normal development and ptilopodous breeds. Furthermore, we have also provided expression patterns of two further genes, *Zic3* and *Zic4*, which were not previously associated with flight feather development (Figure 4). Therefore, we think that the analyses of four genes is sufficiently enough validation, and it would be an intriguing line of research to understand the function of Zic family members in flight feather development. Indeed we have speculated upon this in the discussion.

Other comments:

- The abstract and introduction mention evolutionary implication of the work. While this is an important -and impactful matter, it remains here purely theoretical (as such, data does not allow making strong evolutionary claims). The manuscript should reflect this, and the rather long evolutionary-oriented part of the introduction would be better suited as a discussion paragraph. Also, please introduce information relative to flight feathers prior to the small summary of results.-

This is a good suggestion and we removed the section on flight feathers from the introduction and incorporated the evolutionary considerations into the discussion. We have also removed the first sentence of the abstract that speculated on the evolution of flight feathers. We agree with the referee that the evolutionary considerations, which are important for the paper, are better suited in the discussion.

Please provide literature references on the bio-mechanics of flight feathers to support the argument that they support most of flight forces (even though it is a priori intuitive).

We have included the most recent high-profile reference (Matloff et al, Science 2020 – line 104), further references can be found therein.

- The argumentation is unclear in the first result paragraph: cyclopamine treatment leads to loss of feather buds (and Pitch expression), while no treatment has no effect. It is not possible to conclude from these results that it is the earlier loss of Shh that prevents flight feather formation (a conclusion that can however be made later in the paper). Please clarify.

The point we wanted to make is that the early loss of Shh signalling specifically prevents the formation of one type of feather bud (the flight feathers), but without interfering with the morphogenesis of the feather buds as shown by the normal expression of *Ptch1*.
However, we have re-written the sentence to help clarify. Line numbers 130- 134: '*However, the observation that Ptch1 is still expressed in feather buds of wings treated at HH19 with cyclopamine, suggests that it is the earlier loss of Shh signalling by the polarising region that prevents flight feather bud formation, rather than the loss of Shh signalling within the buds themselves.*'

-Please state more clearly the conclusion in the paragraph describing lineage experiments (i.e., the fact that cells expressing Sim1 lie next to cells originating from the PR region shows that they have been exposed to Shh signaling).

We think this is more of a discussion point, because the data in this section does not show exposure to Shh signalling. However, we have concluded with '*Taken together, the implications of these findings are that Shh signalling induces Sim1 expression and flight feather formation in cells immediately dorsal to the polarising region lineage*' (Lines 287- 289).

-Figure 3: the sequential disappearance of Sim1 expression depending on the timing of

© 2020. Published by The Company of Biologists under the terms of the Creative Commons Attribution License (https://creativecommons.org/licenses/by/4.0/).

cyclopamine administration is one of the strongest experiments of the paper, please present it in a clearer fashion (i.e., provide magnification views in and outside of areas lacking feather buds to clearly show their absence together with that of Sim1 expression).

We attempted to do this but realised that it provided no extra information from what could be seen by enlarging the image manually. In order to help with the visualisation of the data, we have separated the original five figures into nine so that the panels could be enlarged.

-Figure 4: Please explain briefly in the text why chicks treated with cyclopamine at HH19 do not survive beyond hatching. Is this expected?

They died because of failure to close the abdominal wall. We have included this point – line 324.

- *Figure 5 shows a flight feather phenotype obtained in a cyclopamine-treated, then long- term grafted chick individual. A very long and detailed paragraph is dedicated to this result. It has however (understandably) been obtained in only one individual, and given the artifactual nature of grafting experiments, authors should either repeat the experiment for strong conclusions, or significantly shorten writing / tune-down conclusions.*

We have reduced the length of this statement. This was a difficult experiment to perform due to the low numbers of healthy surviving birds, but we think it is an important piece of data because it properly shows the second generation of mature feathers. Although we only show one example like this in the paper, we included the data from five others in the supplementary table 2.

- *It would be nice to see further discussion on the exact role of Shh signaling: does it specify flight feather bud cells, is it necessary for their differentiation (as was previously shown for feather buds in general), or is it required for the spatial patterning of flight feathers?*

We have referenced other studies that discuss the role of Shh in the later differentiation of feathers (Harris et al. 2002; McKinnell et al. 2004; Harris et al. 2005 - line 120), but which is separate to the function we have described here. We have not discussed this in detail because these papers are tangential to our work.

Reviewer 2 Advance Summary and Potential Significance to Field:
The manuscript shows that Sonic Hedgehog (Shh) produced by the ZPA at E3 is required for the formation of the first and second generations of adult flight feathers in chicken. They also identified a group of genes differentially expressed in flight feather buds at E10, after cyclopamine exposure at E3 compared to non-treated wings, group of genes that includes Sim1. The presence of Shh at HH18-HH22 is required for Sim1 expression at E10 in posterior margin of the wing. They also show that Sim1 expression pattern at E10 does not overlap but is close to the ZPA-derived cells.
Although the involvement of Shh in flight feather formation is somehow expected given the fact that Shh specifies all limb structures across the antero-posterior axis and that flight feathers originate from limb lateral-plate, the authors analyse further the mechanism underlying Shh involvement in flight feather formation.

Reviewer 2 Comments for the Author:
One main concern is that text and data do not correlate perfectly. Data appears to be over-interpreted. It is possible that it is just question of writing, I had difficulties to understand the text and did not see data supporting all the conclusions. Here are some exemples. Summary

«Here we show that a positional information gradient of Sonic hedgehog (Shh) signalling in the embryonic chick wing bud specifies the pattern of adult flight feathers in a defined spatial and temporal sequence that reflects their different identities.»
I did not see any data showing this conclusion.

This is a good point and we have restricted mention of the Shh gradient to the discussion where

© 2020. Published by The Company of Biologists under the terms of the Creative Commons Attribution License (https://creativecommons.org/licenses/by/4.0/).

we speculate on our results. From previous work of others and ours, we have strong evidence that graded Shh signalling specifies antero-posterior positional values in digit patterning. Therefore, we think it is warranted that we discuss this in relation to flight feathers that are also patterned in a defined anterior to posterior sequence over the same temporal window. We have also changed the title of the paper to '*Sonic hedgehog specifies flight feather positional information in avian wings*.'

"Figure 3—Shh signalling is interpreted into a spatiotemporal pattern of Sim1 Expression" The data in Figure 3 do not reflect the title of Figure 3. The data in Figure 3 show that Sim1 expression does not overlap but is close to the ZPA-derived cells. I do not see any data showing an interpretation of the spatiotemporal pattern of Shh.

We have changed the phrase to '*The duration of Shh signalling is interpreted into the later spatial pattern of Sim1 expression*' - lines 238 and 786.

SIM1 function in flight feather formation
"Sim1 as a marker of flight feather-forming regions" Does it refer to previous lineage tracing experiments that show where are the flight feather-forming regions?

This refers to the paper that associated Sim1 with the flight feather forming region of the wing (Seki et al, Nat Comms 2017).

Shh is required for Sim1 expression, but it is not clear what could be the function of Sim1 in flight feather formation downstream of Shh. How does Sim1 expression behave upon different concentrations of Shh applied at the anterior margin? Would Sim1 mutant mice provide any additional information?

We have produced new data showing that a polarising region graft duplicates the pattern of flight feather buds (Figure 1a) and *Sim1* expression Figures 5e and 6j). We have also made small grafts of the polarising to mimic the effects of lowering the concentration of Shh signalling. This results in the duplication of digit 1 (Figure 5f) and the *Sim1* expression domain specifically in this digit, thus replicating the effects of a short exposure (equivalent to a low concentration) of Shh signalling when cyclopamine is applied at HH18 (Figure 5a).
Therefore, lowering the concentration of Shh signalling, either endogenously or ectopically, has the same effect on *Sim1* expression and digit patterning. We are unclear what the role of Sim1 is in feather development and hope to examine this in a future study. Although potentially interesting in its own right, we are not sure what *sim1* mutant mice would tell us about the role of Sim1 in integument development that could contribute to our current study.

Additional remarks:
The administration of the Shh inhibitor (cyclopamin) does not target the early limb source of Shh (ZPA), but is delivered in the whole embryo. I was wondering if it was possible to exclude any potential effects of Shh inhibition from other sources (than ZPA) in the embryo.

It would be hard to envisage that Shh from other parts of the embryo could affect feather patterning in the wing. However, our data in Figure 8, in which we transplanted a cyclopamine treated wing bud to a normal embryo and obtained the same defects as treating embryos with cyclopamine, discounts this possibility.

Introduction page 3-4
Other tissues that are not derived from the lateral plate mesoderm, including the nerves and muscles, are duplicated as a secondary consequence, and thus show equivalence (i.e. are not intrinsically different in character and do not carry positional information).
References are needed for this sentence. A recent one for muscle and innervation could be Luxey et al., 2020 PMID: 31697937.

This a very good paper which we have now cited.

Results page 5

© 2020. Published by The Company of Biologists under the terms of the Creative Commons Attribution License (https://creativecommons.org/licenses/by/4.0/).

«The loss of flight feather buds could be interpreted a consequence of missing posterior structures: i.e. digit 3 often does not form in wing buds treated with cyclopamine at HH19» Does it refer to data in the MS or shall it be referenced?

We have now referred to the figure in the paper and also referenced the correct paper.

Figure 1
A schematic of the feather loss upon cyclopamine exposure would be informative.

This is a good suggestion and we have included a schematic.

Figure 4
Here again a schematic of the phenotype would help the reader.

This is also a good suggestion and we have included a schematic in the new Figure 7.

Figure 2 and Figure 3
The panel b of Figure 3 is redundant with the panels f,g of Figure 2. These panels show the same experience: Sim1 expression at E10 after cyclopamine application in early wing buds.

It would seem odd to remove the normal and cyclopamine-treated expression patterns from the middle of the new Figure 4. This figure validates the RNA sequencing across the four conditions and so provides useful comparison that is separate to the new Figure 5.

Figure 5 panel h
Graded Shh signalling between HH18 and HH22 (blue shading—h) specifies the spatiotemporal pattern of Sim1 expression and adult flight feathers in the order that the pattern of skeletal elements is also specified across the antero-posterior axis.
I did not find any experimental argument to say this, since there is no experiment with graded Shh signalling.

This is a speculation based on what we know about how Shh specifies positional information in the chick wing, but we have softened the claim by saying '*Predicted temporal gradient*' – line 857.

Material and methods Page 18
"Embryos were dissected in DMEM and wing buds removed using fine tungsten needles, grafted to the appropriate location of stage-matched host limb buds and held in place with 25 µm platinum pins."
« appropriate location » is a bit vague. A more precise explanation would be appreciated.

We have amended this by saying that they were grafted in place of host wing buds.
We have also added polarising regions were grafted to anterior or posterior margins and referenced a paper (Stainton, 2018) – line 487.

Reviewer 3 Advance Summary and Potential Significance to Field:
The manuscript "A positional information gradient of sonic hedgehog specifies flight feather pattern in the avian wing" by Busby et al. examines the consequences of Shh downregulation on flight feather associated gene expression, and on the development of flight feathers. The authors first show that cyclopamine treatment, which inhibits Shh signalling, causes a loss of flight feathers in the chick. This loss is independent of Shh's role in feather morphogenesis and is not a result of a loss of dorsal/ventral patterning. The loss of flight feather buds is reflected in a loss of flight feathers at hatching. The authors then examine gene expression using RNA-seq and show that expression of Sim1 and members of the Zic family are downregulated in response to cyclopamine treatment. This result is verified through in situ hybridisation. The authors go on to show that the extent of downregulation of Sim1 is dependent on the embryonic stage at which Shh is inhibited, and that the spatial pattern of Sim1 and Shh appear correlated. Finally, the authors show that flight feathers are absent in mature (P66) chicks from those regions of the wing which lacked Sim1 expression. Based on these results, the authors conclude that Shh controls the

© 2020. Published by The Company of Biologists under the terms of the Creative Commons Attribution License (https://creativecommons.org/licenses/by/4.0/).

expression of Sim1 in a spatiotemporally integrated manner, ultimately controlling the patterning of flight feathers.
They further suggest that flight feather patterning co-opted the previously existing Shh gradient which patterns digits in the forelimb and that this transition was crucial to the evolution of wings. The authors corroborate the previous finding that Sim1 is associated with flight feather development (Seki et al. 2017) and advance current knowledge by showing that expression of this gene is affected by Shh inhibition. The data presented do justify the conclusions drawn to an extent, but additional work to investigate the consequence of ectopic Shh expression be necessary to fully support their conclusions. The experiments presented appear well controlled. The manuscript suffers, however, from mislabelling and some lack of clarity, which means that the reader is sometimes unsure as to the experimental conditions for some of the presented data. The work is interesting, but several revisions are required before the manuscript can be recommended for publication.

Reviewer 3 Comments for the Author:

Major Concerns
1) Figure 3 nicely shows the progressive loss of Sim1 expression with earlier treatment by cyclopamine and the correlation between Sim1 expression and transplanted ZPA tissue. Figure 4 and 5 show how this affects the formation of mature flight feathers. However, to determine that Shh is necessary and sufficient for Sim1 expression, and subsequent flight feather development, the authors should show that Shh can induce ectopic Sim1 and flight feathers. This could be done by transplantation of a ZPA from a GFP expressing donor, or cultured Shh expressing cells, to the anterior margin of the limb bud followed by in situ for Sim1, and analysis of mature flight feathers. This would show that Shh is both required for, and capable of inducing, flight feather patterning.

This is a good suggestion and we have included new data showing that polarising region grafts duplicate flight feather buds (Figure 1a) and *Sim1* expression (Figure 5e, Figure 6j) Interestingly, GFP lineage tracing shows the same spatial relationship between the polarising region lineage and *Sim1* as we observed during normal development (Figure 6).

2) The results from the last two experiments seem unclear. Figure 4 appears to show that embryos treated at HH19 form no flight feathers. Supplementary figure 3 is described in the text as showing that later treatment (HH20/21) by cyclopamine leads to formation of flight feathers on distal structures. However, the figure and figure legend imply that the treatment was at HH19.

This is an error and it should have been HH19 and not HH20/21.

Figure 5 suggests that treatment at HH19 will lead to formation of distal primary flight feathers, in agreement with supplementary figure 3, but disagreeing with figure 4. Additionally, in figure 5, transplantation of a limb from an embryo treated with cyclopamine at HH19/20 (although listed in supplementary data as HH20) should lead to formation of only distal primary flight feathers and alular feathers if the schematic is correct, however all secondary flight feathers appear present. This discrepancy in the data needs to be addressed.

The referee is correct to point this out and we are grateful it has been noticed. Embryos treated systemically at HH19 tended to have more severe defects than transplanted wing buds treated at HH19 (now corrected). For ethical considerations we were recommended to reduce the number of these experiments to a minimum due to their unpleasant nature (six birds hatched in total). Three of these had flight feather defects similar to those observed in embryos treated systemically with cyclopamine at HH19. However, the two that survived to p37 and p66 to allow a thorough analysis of their wings had milder defects, and we favour the interpretation that this is due to variability with the cyclopamine treatment that we often observe. The difference in feather pattern in the two experiments (systemic vs. transplants) is still quite minor (present or absent secondaries) that probably reflects a very subtle change in the levels/timing of Shh signalling. However, we were still able to document the important feature this experiment was designed for, which was if feather pattern was affected ventrally or dorsally adjacent to the missing flight feathers.

© 2020. Published by The Company of Biologists under the terms of the Creative Commons Attribution License (https://creativecommons.org/licenses/by/4.0/).

Fig 5h implies that the treatments and transplants was carried out at all the stages listed and the results illustrated, however, treatment was at HH20 or 20/21 for all hatched birds. It is not clear that the diagram in Fig 5h is a schematised hypothesis. The authors should clarify how the experiments were done and further provide their clear result for the different experiments.

The schematic in Figure 9 refers to the average effects on *Sim1* expression and flight feather patterning that we obtain by applying cyclopamine systemically at these different stages, and we have now clarified this in the legend. Unfortunately, no embryos treated with cyclopamine at HH18 survived to hatching, so we made a prediction that alulars would be specified at this time-point because *Sim1* expression was only found in digit 1 at very reduced levels.

'Note that the experiments in which embryos were systemically treated with cyclopamine were used to define the temporal requirement of Shh signalling for Sim1 expression and flight feather development.' In addition, chicks treated at HH18 failed to hatch, thus we are predicting that alulars would be specified at this stage based on Sim1 expression.' Line 867.

3) For the RNA-seq experiment, the controls are not clear for Fig. 2e, j, o and t. The y-axis is labelled Log2 fold expression, however, it is not clear against which control the fold change was calculated. The Bovan's brown leg should be used as the control, but its fold change appears to vary. In contrast to the graphs shown, the figure caption describes the y axis as normalised read-count intensities, however at least two graphs show negative values on in the box plot. The authors should make their controls clear and fix the discrepancy between the figure and the figure legend. This experiment would benefit from qPCR validation of the differences in mRNA transcript levels between the 4 conditions.

This is a mistake on our part and the boxplots describe log2 changes and not log2-fold changes that describe the raw read-count data. The negative values are for those expression values that were between zero and one before log2 transformation (explanation now added to methods section). We think for the purpose of this paper, we are generally documenting absence or presence of gene expression in the forewing/foreleg region, and this is clearly shown by the in situ data. However, we have included qPCR validation for *Sim1* as a supplementary Figure 2. Unfortunately, to investigate expression of other genes by qPCR would require us to collect all the samples again, and we are not sure when we will next be able to obtain Pekin bantam embryos.

Minor Concerns
1) The introduction could benefit from a few more citations, and more considered explanation. For example, the statement "Other tissues that are not derived from the lateral plate mesoderm, including the nerves and muscles, are duplicated as a secondary consequence, and thus show equivalence" is uncited and somewhat unclear.

We have added a reference (Luxey et al 2020). We have amended the phrase about equivalence *(i.e. progenitor cells are not intrinsically different in character and do not carry positional information)*. We have also reference Lewis and Wolpert (1976) for further information. Line 90.

Similarly, in the results section, conclusions are drawn based on results from previous papers which are not fully explained. For example, the end of page 5 and beginning of page 6 - it is not clear why the conversion to more anterior structures should have no impact on feather formation.

It could be expected that the conversion to anterior structures will have an effect on all posterior tissues including flight feathers (unless the flight feathers were specified by signals other than Shh independently of antero-posterior polarity, or patterned at a later stage).

However, it is important to distinguish if the absence of a structure is just a consequence of the loss of progenitor cells, i.e. migrating myoblasts, or due to the transformation of cell identity, i.e. the digits and their progenitor cells. For flight feathers, it is the latter case, thus showing that their progenitor cells are specified with posterior positional information. We have add the phrases: *'The failure of flight feather bud formation could be interpreted as a secondary consequence of the loss of all posterior tissues: i.e. digit 3 often does not form in wing buds*

© 2020. Published by The Company of Biologists under the terms of the Creative Commons Attribution License (https://creativecommons.org/licenses/by/4.0/).

treated with cyclopamine at HH19 (Fig. 1d) (Towers et al. 2011). This is an important consideration because, in the case of the muscles, their absence would be due to the loss of migrating myoblasts into posterior regions of the wing. However, wing bud mesoderm, which differentiates into the dermis, is not lost following cyclopamine exposure, but instead contributes to the development of structures that are anteriorised (i.e. cells that would have contributed to digit 3 contribute to digit 2)'. Lines 135-143. The very unexpected thing about our work is that we see loss of flight feathers even when skeletal elements are still present. Thus flight feathers require slightly longer exposure to Shh for their specification than their associated skeletal elements.

2) Figure captions are confusing as the panel letter is often listed after the described panel, rather than before. This can make it difficult to understand which panel is being described.

We have amended these points.

3) Fig 3h and h' are missing scale bars

We have added these.

4) Unclosed parentheses at the bottom of page 7

We have corrected this.

Second decision letter

MS ID#: DEVELOP/2020/188821

MS TITLE: Sonic hedgehog specifies flight feather positional information in avian wings

AUTHORS: Matthew Towers, Lara Busby, Caitlin McQueen, Cristina Aceituno, Constance Rich, and Marian Ros

I have now received all the referees reports on the above manuscript, and have reached a decision. The referees' comments are appended below, or you can access them online: please go to BenchPress and click on the 'Manuscripts with Decisions' queue in the Author Area.

The overall evaluation is positive and we would like to publish a revised manuscript in Development. Reviewer 2 has a couple of minor points that should be addressed. In addition this referee asks whether the feather phenotype following reduced ZPA grafts correlates with Sim1 expression, if you have these data, adding them would strengthen the study.Please attend to all of the reviewers' comments in your revised manuscript and detail them in your point-by-point response. If you do not agree with any of their criticisms or suggestions explain clearly why this is so.

Reviewer 1

Advance summary and potential significance to field

Authors added a few more experiments to answer R2's comments and provide convincing arguments to answer general and minor comments. They performed appropriate revision of the text and Figures.

Comments for the author

My concerns have been answered / commented in a satisfactory manner

© 2020. Published by The Company of Biologists under the terms of the Creative Commons Attribution License (https://creativecommons.org/licenses/by/4.0/).

Reviewer 2

Advance summary and potential significance to field

The manuscript shows that Sonic Hedgehog (Shh) produced by the ZPA at E3 is required for the formation of the first and second generations of adult flight feathers in chicken. They also identified a group of genes differentially expressed in flight feather buds at E10, after cyclopamine exposure at E3 compared to non-treated wings, group of genes that includes Sim1. The presence of Shh at HH18-HH22 is required for Sim1 expression at E10 in posterior margin of the wing. They also show that Sim1 expression pattern at E10 does not overlap but is close to the ZPA-derived cells. Although the involvement of Shh in flight feather formation is somehow expected given the fact that Shh specifies all limb structures across the antero-posterior axis and that flight feathers originate from limb lateral-plate, the authors analyse further the mechanism underlying Shh involvement in flight feather formation.

Comments for the author

The revised MS has improved compared to the initial MS. Schematics are helpful and most of the notion of Shh gradient associated with flight feather (not supported by experiments) has disappeared in the result sections.
A few comments:
Shh grafts would have been more appropriate than ZPA grafts to make the converse of cyclopamin experiments.
One missing experiment is the feather phenotype following reduced ZPA grafts to be correlated with ectopic Sim1 activation.
The fact that Figure 1A (ZPA grafts) is mentioned in the introduction is a bit confusing. If this is the first time that feather duplication is observed after ZPA grafts, the experiment should be mentioned in the result section, If not references should be added in the introduction.

Line 258-261
« In day 10 wings, Sim1 expression is specifically duplicated along the anterior margin of the additional digit 1 (asterisk, Fig. 5F), mimicking the effect of applying cyclopamine at HH18 to attenuate endogenous Shh signalling (Fig. 5A). »
The sentence is misleading. reduced ZPA graft (Shh GOF) application mimics a cyclopamine (Shh LOF) effect. This is better explained in the response to reviewer2

Lines 262-265 These findings reveal that Shh signalling from the polarising region between HH18 and HH22 specifies the later pattern of Sim1 expression in a defined spatial and temporal sequence, which can be replicated by an ectopic source of Shh signalling at the anterior margin of the wing bud in a dose-dependent manner.
ZPA is a source of Shh but not only. It would be more appropriate to precise, ZPA known to produce Shh.

Reviewer 3

Advance summary and potential significance to field

This manuscripts examines the consequences of Shh downregulation on flight feather associated gene expression, and on the development of flight feathers. The authors first show that cyclopamine treatment, which inhibits Shh signalling, causes a loss of flight feathers in the chick. This loss is independent of Shh's role in feather morphogenesis and is not a result of a loss of dorsal/ventral patterning. The loss of flight feather buds is reflected in a loss of flight feathers at hatching. The authors then examine gene expression using RNA-seq and show that expression of Sim1 and members of the Zic family are downregulated in response to cyclopamine treatment. This result is verified through in situ hybridisation. The authors go on to show that the extent of downregulation of Sim1 is dependent on the embryonic stage at which Shh is inhibited, and that the spatial pattern of Sim1 and Shh appear correlated. Finally, the authors show that flight feathers are absent in mature (P66) chicks from those regions of the wing which lacked Sim1 expression. Based on these results, the authors conclude that Shh controls the expression of Sim1 in a

© 2020. Published by The Company of Biologists under the terms of the Creative Commons Attribution License (https://creativecommons.org/licenses/by/4.0/).

spatiotemporally integrated manner, ultimately controlling the patterning of flight feathers. They further suggest that flight feather patterning co-opted the previously existing Shh gradient which patterns digits in the forelimb and that this transition was crucial to the evolution of wings. The authors corroborate the previous finding that Sim1 is associated with flight feather development (Seki et al. 2017) and advance current knowledge by showing that expression of this gene is affected by Shh inhibition.

Comments for the author

The authors have adequately addressed my concerns. I recommend publishing this contribution, in its current form.

Second revision

Author response to reviewers' comments

Reviewer 2 Comments for the author

A few comments:
Shh grafts would have been more appropriate than ZPA grafts to make the converse of cyclopamin experiments.

The reason we chose ZPA grafts is that they produce the same effects on feather pattern as Shh-grafts (see Duprez et al, Mech Dev 1999, v82: p151 for example). However, the duplications obtained with ZPA grafts are much more reliable than those obtained using Shh-grafts.

One missing experiment is the feather phenotype following reduced ZPA grafts to be correlated with ectopic Sim1 activation.

At day 14 the alular flight feather buds are still yet to form (see Figure 1a), therefore we are unable to tell if they are duplicated by ZPA grafts. Unfortunately, we were not granted ethical approval to look at duplicated wings that were older than day 14. We don't think this data is essential for our manuscript as the study of flight feathers in normal development is more informative than when they are ectopically duplicated. We have added the sentence: 'However, we were not able to determine if flight feather buds were duplicated, because at day 14, alular feather buds have still not formed (See Figure 1a), and due to the nature of the experiments, we could not obtain ethical approval to look at older specimens.' (Lines 262-264). However, we think that it is a reasonable inference to assume that alulars will form.

The fact that Figure 1A (ZPA grafts) is mentioned in the introduction is a bit confusing. If this is the first time that feather duplication is observed after ZPA grafts, the experiment should be mentioned in the result section, If not references should be added in the introduction.

This result has been documented before, although not explicitly stated (Riddle, et al 1993). Therefore, we think it is appropriate to reference this paper.

Line 258-261
« In day 10 wings, Sim1 expression is specifically duplicated along the anterior margin of the additional digit 1 (asterisk, Fig. 5F), mimicking the effect of applying cyclopamine at HH18 to attenuate endogenous Shh signalling (Fig. 5A). »
The sentence is misleading. reduced ZPA graft (Shh GOF) application mimics a cyclopamine (Shh LOF) effect. This is better explained in the response to reviewer2

© 2020. Published by The Company of Biologists under the terms of the Creative Commons Attribution License (https://creativecommons.org/licenses/by/4.0/).

We have incorporated the sentence from the previous response 'Therefore, lowering the concentration of Shh signalling, either endogenously or ectopically, has the same effect on Sim1 expression' (Lines 260-262)

Lines 262-265
These findings reveal that Shh signalling from the polarising region between HH18 and HH22 specifies the later pattern of Sim1 expression in a defined spatial and temporal sequence, which can be replicated by an ectopic source of Shh signalling at the anterior margin of the wing bud in a dose-dependent manner.
ZPA is a source of Shh but not only. It would be more appropriate to precise, ZPA known to produce Shh.

We have replaced 'ectopic source of Shh signalling' with 'polarising region grafts' (Line 267).

Third decision letter

MS ID#: DEVELOP/2020/188821

MS TITLE: Sonic hedgehog specifies flight feather positional information in avian wings

AUTHORS: Matthew Towers, Lara Busby, Caitlin McQueen, Cristina Aceituno, Constance Rich, and Marian Ros
ARTICLE TYPE: Research Article

I am happy to tell you that your manuscript has been accepted for publication in Development, pending our standard ethics checks.

© 2020. Published by The Company of Biologists under the terms of the Creative Commons Attribution License (https://creativecommons.org/licenses/by/4.0/).
